# Targeting SARS-CoV-2 Non-Structural Proteins: A Blueprint for Next-Generation Small-Molecule Coronavirus Antivirals

**DOI:** 10.3390/pharmaceutics18060693

**Published:** 2026-06-02

**Authors:** Exequiel O. J. Porta, Dana F. AlKharboush, Lauren Jackson, Felix Pang, Aylin Darin, Joy Louka, Mohammed Quamruzzaman, Xinyue Shi, Geoffrey Wells, Frank Kozielski

**Affiliations:** 1 UCL School of Pharmacy, University College London, London WC1N 1AX, UK; 2 Department of Pharmaceutical Chemistry, Faculty of Pharmacy, King Abdulaziz University, Jeddah 22252, Saudi Arabia

**Keywords:** SARS-CoV-2, non-structural protein (Nsp), direct-acting antivirals, main protease, 3C-like protease (3CLpro/Mpro), papain-like protease (PLpro), RNA-dependent RNA polymerase (RdRp), Nsp14/Nsp16 RNA capping enzymes, antiviral resistance, pan-coronavirus preparedness

## Abstract

The SARS-CoV-2 non-structural proteome remains the most clinically validated and strategically important landscape for direct-acting small-molecule antiviral drug discovery. The success of inhibitors targeting the main protease (M^pro^, Nsp5) and RNA-dependent RNA polymerase (RdRp, Nsp12) has firmly established viral replication enzymes as tractable, druggable, and therapeutically relevant targets, while setting clear benchmarks for translational antiviral development. Building on this foundation, a second wave of non-structural protein (Nsp) targets has emerged with increasing translational promise, including the papain-like protease (PL^pro^), the bifunctional Nsp14 proofreading and capping machinery, Nsp16 2′-O-methyltransferase, Nsp13 helicase, and Nsp15 endoribonuclease. In parallel, additional components such as Nsp1 and the Mac1 domain of Nsp3 continue to expand the antiviral design space, although they remain at earlier stages of chemical validation. In this review, we comprehensively assess SARS-CoV-2 non-structural proteins through a medicinal chemistry and translational lens, with an emphasis on structural tractability, mechanism of action, quality of chemical matter, cellular and in vivo antiviral evidence, evolutionary conservation, resistance liabilities, and developability. Particular attention is given to the features that distinguish tool compounds from genuinely actionable leads and to the opportunities for rational combination regimens that extend beyond first-generation protease- and polymerase-centred therapy. Collectively, the non-structural proteome offers the strongest foundation for next-generation and potentially broader-spectrum coronavirus antivirals with improved resilience to viral evolution.

## 1. Introduction

The emergence of severe acute respiratory syndrome coronavirus 2 (SARS-CoV-2) in late 2019 triggered the coronavirus disease 2019 (COVID-19) pandemic, creating a global health crisis of unprecedented scale and exposing major vulnerabilities in the world’s capacity to respond rapidly to novel viral threats [1,2]. Although coronaviruses replicate with higher fidelity than many RNA viruses because of Nsp14-mediated proofreading, SARS-CoV-2 nevertheless showed substantial evolutionary plasticity, generating successive variants of concern that challenged public health responses and eroded the efficacy of first-generation vaccines and antibody therapies [3,4,5]. Although vaccination has been central to reducing severe disease and mortality, the pandemic also underscored the indispensable role of direct-acting antivirals (DAAs) as a complementary pillar of infectious disease control [6,7,8]. Small-molecule antivirals remain essential for treating established infections, reducing the risk of progression in high-risk patients, and providing therapeutic options for individuals who cannot be fully protected by vaccination, including the immunocompromised [9]. They are also critical for the long-term management of coronavirus infections in an endemic setting, where breakthrough infections, viral evolution, and uneven immune protection remain ongoing challenges [10,11].

The case for continued antiviral drug discovery extends well beyond that of SARS-CoV-2. COVID-19 was the third major pathogenic β-coronavirus spillover into humans in the twenty-first century, following SARS-CoV and Middle East respiratory syndrome coronavirus (MERS-CoV), reinforcing the view that coronavirus emergence is not an isolated event but a recurring biological threat [12]. This reality has driven a strategic shift from a purely reactive, outbreak-specific model of drug discovery toward a broader preparedness framework aimed at developing antiviral agents with utility against future variants and, ideally, across related coronaviruses [13]. In this context, the search for variant-resilient, orally deployable, and mechanistically diverse DAAs is no longer simply a response to the last pandemic, but a central component of future pandemic preparedness [14,15,16,17,18], supported by global initiatives such as the “100 Days Mission”, which aims to radically accelerate medical countermeasure development for future outbreaks [19,20].

**Figure 1:**
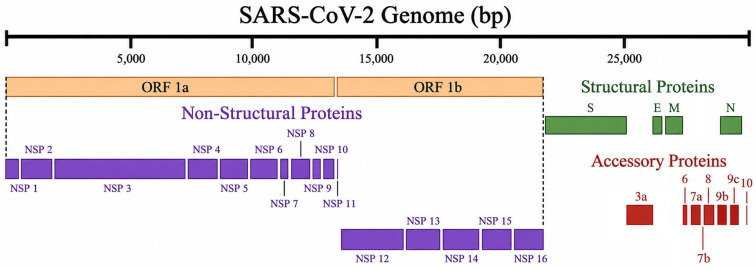
Genome organisation of SARS-CoV-2. The ~30 kb positive-sense RNA genome encodes Nsps within ORF1a/ORF1b and, in the 3′ region, the structural (S, E, M, N) and accessory proteins (e.g., 3a, 6, 7a, 7b, 8, 9b, 9c, 10). Cap-dependent translation initiates at ORF1a to produce polyprotein pp1a (Nsp1–Nsp11). A programmed −1 ribosomal frameshift at the ORF1a/ORF1b junction enables read-through to generate pp1ab, the longer polyprotein comprising Nsp1–Nsp16. The Nsps are liberated by viral proteases (PL^pro^ and M^pro^).

Among the proteins encoded by SARS-CoV-2, the non-structural proteins (Nsps) represent the most clinically validated and mechanistically tractable landscape for small-molecule antiviral intervention [21,22]. Generated by proteolytic processing of the viral polyproteins pp1a and pp1ab [23], the Nsps assemble into the replication–transcription complex (RTC), which orchestrates viral genome replication, RNA processing, proofreading, and transcript maturation (Figure 1) [12]. This proteome contains the most important catalytic functions in the coronavirus life cycle, including proteolysis, RNA synthesis, helicase activity, RNA capping, and RNA quality control. From a drug discovery perspective, these functions are especially attractive because they are essential for viral fitness and often lack close human counterparts at the relevant drug-binding surfaces, enabling a clearer path toward selective small-molecule inhibition [18,22,24,25]. The first generation of clinically successful SARS-CoV-2 antivirals strongly validated this strategy: inhibition of the main protease (M^pro^, Nsp5) and the RNA-dependent RNA polymerase (RdRp, Nsp12) established that viral Nsps can support rapid translation from target selection to approved therapy [26,27,28].

At the same time, the limitations of first-generation agents have made it clear that the current therapeutic arsenal remains incomplete [28]. The intravenous administration requirements of Remdesivir constrained its earliest clinical utility [29,30], while the oral combination Nirmatrelvir/Ritonavir demonstrated the power of protease inhibition but also highlighted the practical burden imposed by pharmacokinetic boosting and drug–drug interactions (DDI) [26,27,31]. Likewise, the broader experience with approved and emergency-use antivirals emphasized the need for additional chemotypes, higher resistance barriers, improved convenience, and more flexible combination strategies suitable for early outpatient use. These realities sharpen the rationale for expanding beyond the initial benchmark targets and systematically assessing which additional Nsps are now sufficiently mature to support the next generation of orally deployable antiviral regimens.

In this review, we examine the SARS-CoV-2 non-structural proteome through a target-prioritised medicinal chemistry and translational framework, focusing on proteins that offer the strongest current or emerging rationale for small-molecule intervention. Structural and accessory proteins are mentioned here only for a broader biological context and are discussed in detail in the companion review [32]. For each non-structural target, we assessed the biological function, structural tractability, assay modalities, quality of chemical matter, resistance liabilities, and key determinants of developability, with particular emphasis on the translational properties most relevant to clinical utility, including oral exposure, pharmacokinetic robustness, and suitability for short-course outpatient therapy. Herein, we focus exclusively on small molecules with demonstrated biological activity. To reflect the unequal maturity of the current antiviral landscape, the discussion is organised into three priority tiers: clinically validated benchmark targets, near-term translational priorities, and early stage but strategically important opportunities. This framework is intended not only to catalogue progress but also to distinguish which Nsps are most likely to support the next generation of variant-resilient and potentially broader-spectrum coronavirus therapeutics.

## 2. Clinically Validated Benchmark Nsp Targets

The clearest proof that the SARS-CoV-2 non-structural proteome can deliver clinically useful small-molecule antivirals comes from two targets: M^pro^ and RdRp. Together, these enzymes underpin the first clinically successful DAA strategies against COVID-19 and establish a practical benchmark against which other Nsp targets should be assessed. Beyond their central roles in viral proteolysis and RNA synthesis, they define the key translational features that distinguish a biochemically tractable target from a clinically actionable one, including robust enzymatic validation, structurally accessible binding sites, reproducible antiviral activity in cellular systems, pharmacokinetic properties compatible with early outpatient use, and a resistance profile that can support durable therapeutic benefit. Therefore, we begin with M^pro^ and RdRp as the reference points for target maturity in the SARS-CoV-2 antiviral landscape, using the lessons learned from these validated systems to inform the evaluation of additional Nsps discussed in the following sections.

### 2.1. Main Protease Nsp5 (3CL^pro^ or M^pro^)

#### 2.1.1. Biology and Rationale

Nsp5, the main protease, is indispensable for replication of coronaviruses. It performs 11 cleavage events on the polyproteins pp1a/pp1ab, cutting at consensus Leu-Gln↓(Ser/Ala/Gly) sequences to release mature Nsps (from Nsp4 to Nsp16) [33]. In other words, by cleaving the viral polyprotein at many sites, M^pro^ releases the mature replication machinery. Inhibition of M^pro^ halts viral RNA synthesis and thus virus replication [34]. In addition, there is no human equivalent of this cysteine protease with the same specificity, making it a top-priority antiviral target [35]. Indeed, the first widely approved oral SARS-CoV-2 antiviral regimen, Nirmatrelvir/Ritonavir (Paxlovid), targets M^pro^. The validation of M^pro^ occurred even earlier during the SARS-CoV-1 outbreak when peptidomimetic inhibitors were shown to inhibit the virus [36].

#### 2.1.2. Assays and Structural Biology

M^pro^ has been extensively characterised structurally. Within weeks of SARS-CoV-2 genome release, the crystal structure of M^pro^ was determined (February 2020) in complex with a peptide inhibitor (N3) [37]. Over 300 M^pro^ structures are now in the Protein Data Bank (PDB), including many co-crystals with inhibitors of diverse chemotypes. This abundance of structural data has guided structure-based drug design (SBDD) research. Enzymatic assays for M^pro^ use fluorescent peptides (e.g., DABCYL-KTSAVLQ↓SGFRKME-EDANS) that generate a signal when cleaved [38,39]. Cell-based assays include viral replication assays in Vero or human cells, often measuring viral RNA or cytopathic effects [40]. Given the central role of M^pro^, compounds typically show a strong correlation between enzyme IC_50_ and antiviral EC_50_, although cell permeability can modulate the latter.

#### 2.1.3. Chemical Matter and SAR

M^pro^ drug discovery has been highly fruitful (full clinical benchmarks are summarised in Section 2.3). Key classes of chemical matter include peptidomimetic covalent inhibitors, non-peptidic covalent inhibitors, and non-covalent inhibitors.

**Figure 2:**
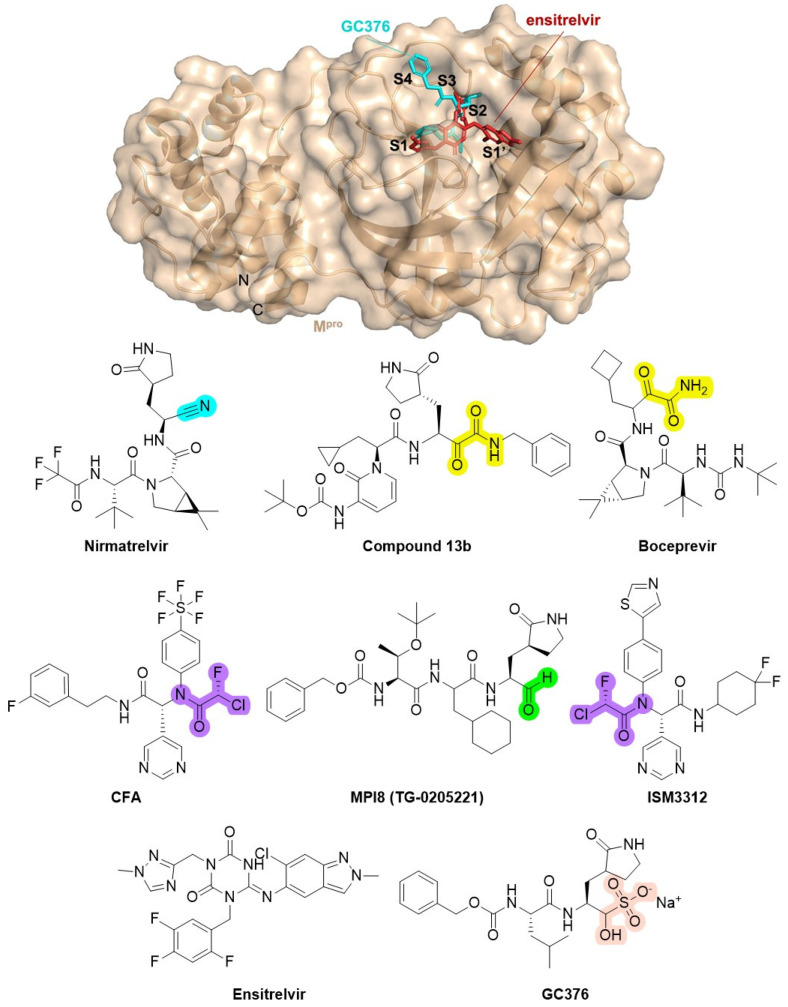
**Top:** Crystal structure of SARS-CoV-2 M^pro^ (beige) in complex with Ensitrelvir (red; PDB ID 7VU6), with the ligand GC376 (cyan; PDB ID 9EET) superposed for comparison. **Bottom:** Representative chemotypes for SARS-CoV-2 M^pro^ inhibition, including Nirmatrelvir [43], Compound 13b [42], Boceprevir [49], chlorofluoroacetamide (CFA) inhibitors [47], ISM3312 [48], MPI8/TG-0205221 [36,50], Ensitrelvir [51], and GC376 [49]. Electrophilic warheads responsible for covalent or reversible covalent engagement of Cys145 are highlighted to distinguish α-ketoamide/ketoamide (yellow), aldehyde (green), nitrile (blue), bisulfite adduct/prodrug group (pink), and chlorofluoroacetamide (purple) mechanisms.

Peptidomimetic covalent inhibitors: These often incorporate a glutamine surrogate at P1 (to bind the S1 pocket) and an electrophile (warhead) to covalently modify the catalytic Cys145 [41]. Early covalent peptidomimetic inhibitors include α-ketoamides, such as Compound 13b, and related ketoamide scaffolds, such as the repurposed hepatitis C virus protease inhibitor Boceprevir (Figure 2). In these compounds, the electrophilic carbonyl warhead is positioned for nucleophilic attack by the catalytic Cys145 thiol, forming a reversible thiohemiketal adduct [42]. X-ray structures confirmed these covalent mechanisms (PDB ID 6Y2F for an aldehyde inhibitor, PDB ID 6WNP for Boceprevir). The peptidomimetics spanned P4 to P1′ positions, optimising interactions in subsites S4–S1′. Notably, Pfizer built on these leads to develop Nirmatrelvir, a peptidomimetic with a nitrile warhead [43,44,45]. Nirmatrelvir (PF-07321332) is a tripeptide-derived, covalently reversible inhibitor (forming a thioimidate with Cys145) incorporating a P2 cyclopropyl glutamine mimic and a P3 tert-butylserine to optimise both potency and metabolic stability [43]. Nirmatrelvir’s oral bioavailability was achieved in part by its unique non-peptidic P3 capping (a rigid bicyclic structure) that reduces polarity. When administered with the PK booster Ritonavir, it maintains plasma concentrations inhibitory to SARS-CoV-2. In the EPIC-HR trial, oral Nirmatrelvir/Ritonavir (Paxlovid) showed 89% reduction in progression to severe COVID-19 in high-risk patients, validating M^pro^ as a clinical target [46].

Non-peptidic covalent inhibitors: Efforts have been made to develop smaller molecules beyond peptide-like structures (Figure 2). Some successes include covalent inhibitors with alternative warheads, such as chlorofluoroacetamides (CFA, irreversible warhead targeting Cys145) [47,48]. For instance, ISM3312 is an orally available irreversible covalent M^pro^ inhibitor (CFA warhead) discovered through generative-AI-guided, structure-based optimisation. It represents one of the earliest clinical-stage examples of generative-AI-enabled COVID-19 drug design and shows nM, pan-coronavirus activity across SARS-CoV-2 variants and endemic human coronaviruses, with efficacy in human airway organoids and multiple in vivo models [48]. It retains activity against Nirmatrelvir-resistant M^pro^ mutants, exhibits only moderate resistance on serial passaging, and its antiviral effect is largely independent of P-glycoprotein (P-gp) efflux, supporting sequential or combination use with Nirmatrelvir to curb resistance and strengthen pandemic preparedness. Another chemotype is GC376 (a dipeptidyl compound occupying S1, S2, S3, and S4 pockets). GC376 is a bisulfite adduct prodrug of the corresponding aldehyde inhibitor. Following release of the active aldehyde species, the electrophilic carbonyl reacts reversibly with the catalytic Cys145 thiol to form a reversible covalent thiohemiacetal adduct. Therefore, GC376 is better described as an aldehyde-prodrug/reversible covalent M^pro^ inhibitor rather than as a bisulfite adduct directly engaging Cys145 [49]. It was originally an animal coronavirus drug candidate for feline infectious peritonitis virus and showed activity against SARS-CoV-2 but was not pursued clinically, likely due to its parenteral administration. Finally, aldehyde-containing M^pro^ inhibitors represent a separate reversible covalent class that forms thiohemiacetal adducts with Cys145. Pharmacophore-based designs have yielded aldehyde-containing compounds such as MPI8 (also known as TG-0205221), a pyridinone covalent inhibitor, with sub-μM antiviral activity and good metabolic stability [36,50]. Generally, smaller covalent inhibitors can benefit from better permeability but must carefully orient the warhead for selective reaction with Cys145.

Non-covalent inhibitors: These are attractive for avoiding potential off-target reactivity, although achieving high potency is more challenging. A notable non-covalent series is Shionogi’s Ensitrelvir (S-217622) (Figure 2) [51]. Ensitrelvir is a triazole-containing, non-peptidic inhibitor that binds to M^pro^. It was optimised for oral delivery and potency, and remarkably, it became the first orally active, non-covalent, non-peptidic M^pro^ inhibitor to be approved (approved in Japan in 2022–2023). Ensitrelvir is effective against SARS-CoV-2 variants and has shown significant antiviral efficacy in clinical trials (faster viral clearance) [52]. Structurally, Ensitrelvir occupies the S1, S1′, and S2 pockets similarly to peptidic inhibitors but relies on tight binding rather than covalent anchoring.

#### 2.1.4. PK/PD and Clinical Data

Nirmatrelvir has a half-life suitable for twice-daily dosing when boosted by Ritonavir [53]. Paxlovid’s clinical performance is well documented: in non-hospitalised high-risk patients, it reduced COVID-19 hospitalization or death by 88–89% compared to placebo [7]. It retains activity against all major variants (Alpha through Omicron) because M^pro^ is conserved (only a few variant polymorphisms occur at non-critical sites) [54,55]. Ensitrelvir, given once daily, also showed significant reduction in viral RNA load and symptom relief time in Phase 3 trials (for example, faster clearance of infectious virus by 1–2 days) [52]. Ensitrelvir received approval in Japan based on these data (among others). The safety profiles of M^pro^ inhibitors have been favourable: the main side effects of Nirmatrelvir are related to Ritonavir’s interactions, and Ensitrelvir’s trials reported mostly mild adverse events such as taste disturbance [34].

#### 2.1.5. Resistance

As anticipated, M^pro^ inhibitors face potential resistance [54]. However, the clinical use of Paxlovid has led to reports of rebound in some patients, but not clearly linked to resistant mutants [56]. Nonetheless, resistance can be selected in vitro [57]. Experiments have identified mutations such as P132H in M^pro^ that can confer resistance to Nirmatrelvir (this mutation is seen in Omicron, though it remains largely susceptible) [58]. Other laboratory-generated mutations include G15S and K90R, which modestly reduce inhibitor binding [59]. Structural studies of these mutant proteases bound to Nirmatrelvir indicate that the binding mode is mostly retained.

Serial passaging of SARS-CoV-2 with Ensitrelvir selected M^pro^ substitutions M49L and E166A, which confer reduced drug sensitivity. Each mutation impaired viral growth in vitro in the absence of the drug; however, their combination largely abrogated Ensitrelvir’s suppressive effect [60]. Crucially, because M^pro^ function is essential, most resistance mutations impose a fitness cost on the virus; therefore, high-level resistance may emerge slowly. To proactively address this, combination therapies (M^pro^ inhibitor + another antiviral) or next-generation M^pro^ inhibitors effective against resistant variants are being pursued [61].

#### 2.1.6. Development Status

In addition to Paxlovid, which is authorised globally, and Ensitrelvir, which is approved in Japan, several M^pro^ inhibitors have achieved regional regulatory milestones. Notably, the Ritonavir-boosted covalent inhibitor Simnotrelvir (Xiannuoxin) received regular approval in China in mid-2024, while the non-boosted agent Leritrelvir (RAY1216) was granted conditional approval in the same market (see Table 1 in Section 2.3). Beyond these approved therapies, a second generation of M^pro^-targeted small molecules is actively advancing through clinical pipelines; for example, Pfizer’s new candidate PF-07817883 is currently undergoing evaluation [62]. Additionally, intranasal M^pro^ inhibitors are being investigated for prophylaxis, aiming to achieve early infection control via direct delivery to the respiratory tract [63]. To broaden therapeutic efficacy and mitigate resistance, these M^pro^ agents are also frequently evaluated in clinical trials in combination with polymerase inhibitors, such as Remdesivir analogues (*vide infra*) [64]. As of early 2026, Paxlovid remains the dominant, broadly approved oral antiviral for outpatient COVID-19 treatment across most international markets. Ensitrelvir, which offers a streamlined once-daily dosing regimen relative to the twice-daily requirement of Paxlovid, represents a highly promising alternative, though it has not yet secured widespread global regulatory approval.

#### 2.1.7. Challenges and Opportunities

M^pro^ drug discovery is a success story, but challenges remain: Ritonavir boosting for Paxlovid introduces drug–drug interaction issues [65]; thus, non-boosted inhibitors (such as Ensitrelvir) are desirable. There is also interest in pan-coronavirus M^pro^ inhibitors. M^pro^ is conserved among β-coronaviruses (e.g., SARS-CoV-1 and MERS M^pro^ share 96% and 50% identity with SARS-CoV-2 M^pro^, respectively) [55]. Many SARS-CoV-2 inhibitors also inhibit SARS-CoV-1 M^pro^; some even inhibit MERS M^pro^ albeit weaker [48]. Broad-spectrum M^pro^ inhibitors could be stockpiled for future outbreaks. Another opportunity is structure-enabled drug design: with artificial intelligence (AI) tools and abundant crystallographic data, designing inhibitors de novo (even via algorithms) is increasingly feasible; ISM3312 exemplifies this trajectory. Finally, M^pro^ inhibitors may be combined with host-directed agents (e.g., TMPRSS2 inhibitors or anti-inflammatories) to enhance outcomes for severe COVID-19 [63]. In conclusion, M^pro^ is the most clinically advanced SARS-CoV-2 protein target. Continued optimisation focuses on dosing convenience, variant resistance-proofing, and expanding the arsenal to ensure long-term efficacy against SARS-CoV-2 and related viruses.

### 2.2. RNA-Dependent RNA Polymerase Complex (RdRp, Nsp12, with Nsp7/Nsp8 Cofactors)

#### 2.2.1. Biology and Rationale

The RNA-dependent RNA polymerase (RdRp, Nsp12) is the core enzyme that copies the viral RNA genome [66]. It is essential for viral replication, making it a validated drug target, exemplified by Remdesivir (full clinical benchmarks for RdRp are summarised in Section 2.3). However, RdRp does not act alone: for optimal function, it forms a complex with Nsp7 and Nsp8 as cofactors [66]. Nsp7 and Nsp8 serve as processivity factors or primase elements, helping the polymerase to initiate and elongate RNA strands [67]. In fact, the active replicase is a multisubunit machine called the replication–transcription complex (RTC), transiently comprising RdRp, Nsp7/Nsp8 (polymerase cofactors), helicase, bifunctional Nsp14, and Nsp10 as a stimulator for Nsp14 and Nsp16 [66]. In this section, we focus on RdRp and its immediately associated proteins Nsp7 and Nsp8, which have been structurally characterised as a complex.

#### 2.2.2. Assays and Structural Biology

The structure of the SARS-CoV-2 RdRp/Nsp7/Nsp8 complex was solved early in the pandemic via cryo-electron microscopy (cryo-EM) at 2.9 Å resolution [68]. It revealed the canonical right-hand polymerase architecture (palm, fingers, and thumb domains) and an N-terminal extension with a Nidovirus RdRp-associated nucleotidyltransferase (NiRAN) subdomain that may contain nucleotidyltransferase activity. Nsp7 and Nsp8 form a collar around RdRp, with Nsp8 also protruding to guide the exiting RNA. The structures of Nsp12 bound to RNA and incoming nucleotides (with Remdesivir or other analogues) have also been determined [69]. These structures illuminate how nucleotide analogue drugs work. Enzymatic assays for RdRp use short RNA templates and primers to measure the incorporation of nucleotides (radioactive or fluorescent) [70]. Cell-based assays rely on measuring virus replication, as polymerase inhibition stops viral RNA production (often reflected in viral RNA load by qPCR) [71].

#### 2.2.3. Chemical Matter—Nucleoside Analogues

**Figure 3:**
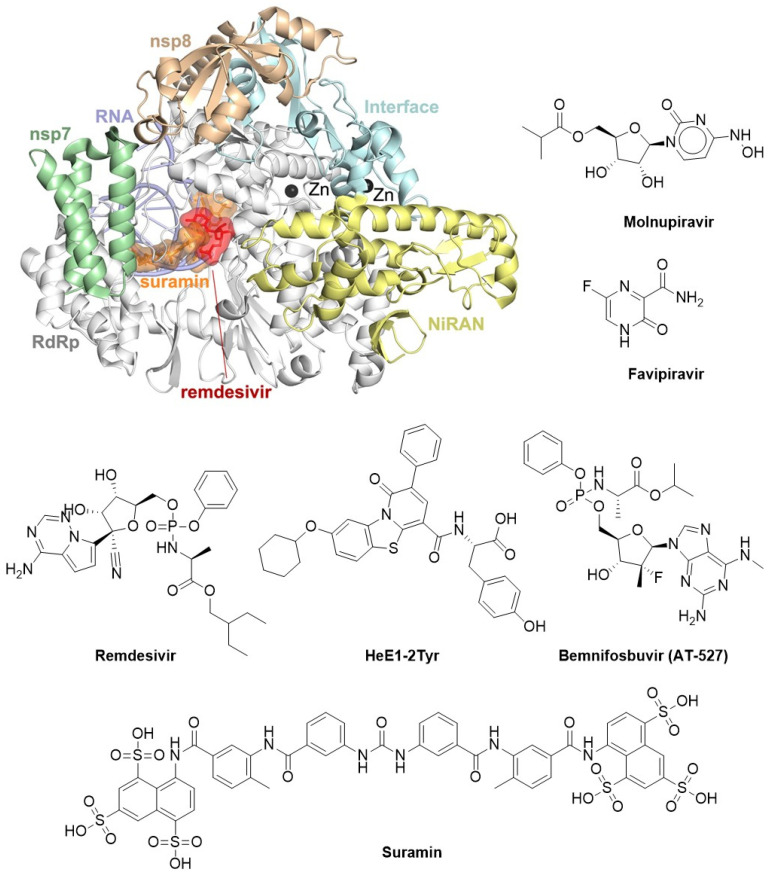
**Top left:** Cryo-EM structure of the SARS-CoV-2 Nsp12–Nsp7–Nsp8 polymerase complex bound to RNA and remdesivir (PDB ID 7BV2), with suramin (orange; PDB ID 7D4F) superposed for comparison. Nsp7 is shown in green, Nsp8 in beige, the Nsp12 RdRp domain in grey, the Nsp12 NiRAN domain in yellow, the Nsp12 interface domain in cyan, RNA in purple, and Zn ions in dark grey. Remdesivir is shown in red. **Top right and bottom:** Representative inhibitors of the SARS-CoV-2 polymerase, including Remdesivir [73], Molnupiravir [78], Favipiravir [79], Suramin [80], HeE1-2Tyr [81], and Bemnifosbuvir (AT-527) [82].

Historically, viral polymerases have been frequently targeted by nucleoside analogues that cause chain termination or lethal mutagenesis. Remdesivir (Figure 3) was originally developed against the Ebola virus and quickly repurposed for SARS-CoV-2 [72]. Remdesivir is a prodrug of an adenosine analogue. Once activated to its triphosphate form, it is incorporated into viral RNA by RdRp and causes delayed chain termination (after adding a few more nucleotides) [73]. Remdesivir has moderate potency (in cell assays EC_50_ ~1 μM) [74] but showed efficacy in hospitalised patients by shortening recovery time [75]. It received full approval for intravenous (IV) use in late 2020 [76]. Chemically, Remdesivir includes a 1′-cyano modification on ribose that confers selectivity for viral polymerase and resists proofreading to some extent [77]. However, the requirement for IV infusion limits its outpatient use.

A major advance was the development of Molnupiravir (EIDD-2801), an oral prodrug. Molnupiravir’s active metabolite, N^4^-hydroxycytidine triphosphate (NHC-TP), is incorporated by RdRp and can base-pair with G or, after tautomerisation, with A, causing C→U and G→A transition mutations that accumulate to drive lethal mutagenesis, also called “error catastrophe” [78]. It demonstrated a 30% reduction in hospitalisation risk in trials and was authorised for emergency use [83]. Molnupiravir’s mutagenesis mechanism raises concerns about potential incorporation into host DNA (e.g., in rapidly dividing cells), but the short treatment duration mitigates this. Given the modest efficacy signals and theoretical risks, uptake has been limited. In the European Union (EU), the Committee for Medicinal Products for Human Use (CHMP) recommended refusal in February 2023, and the sponsor withdrew its marketing authorisation (MA) application in June 2023.

Favipiravir (a purine base analogue) showed broad activity against RNA viruses including coronaviruses, in vitro, but did not conclusively benefit COVID-19 patients in clinical trials [79]. Ribavirin, a general antiviral, was similarly not very effective [84,85]. There are ongoing efforts to develop improved nucleoside analogues: for example, Bemnifosbuvir (AT-527) is a guanosine analogue prodrug that has reached Phase 2 trials [82]. Galidesivir (adenosine analogue) and Rhodanine analogues are also being investigated [24,86]. Importantly, the proofreading exoribonuclease domain (ExoN) of Nsp14 can excise some incorporated analogues (like Ribavirin or 5-Fluorouracil), but analogues like Remdesivir partially evade this [87]. Combining a nucleoside analogue with an ExoN inhibitor (see Nsp14 section) is a theoretical strategy to boost effectiveness.

#### 2.2.4. Chemical Matter—Non-Nucleoside Inhibitors

Non-nucleoside inhibitors have been challenging to identify for coronaviruses. Unlike HIV or HCV polymerases, which present druggable allosteric sites, the coronavirus RdRp forms a compact Nsp12–Nsp7/Nsp8 complex with few obvious small-molecule pockets [81]. Nonetheless, some studies have reported allosteric inhibitors that bind RdRp outside the active site. A report described Suramin (a large polyanionic drug) binding to RdRp and Nsp13, blocking polymerase activity [80]. Suramin showed in vitro activity but is a bulky, polysulfonated repurposed molecule (bearing six sulfonate groups) with well-known target promiscuity (Figure 3) [81]. High-throughput screening has yielded several chemotypes, but most remain at the hit-to-lead stage, and to date, none approach the potency or breadth of leading nucleoside analogues [88,89]. A recent cryo-EM/mechanistic study established HeE1-2Tyr as a bona fide non-nucleoside RdRp inhibitor: it competitively displaces RNA and binds as a stack of three molecules in the RNA-binding channel, stabilised by an “arginine bracket” (Arg555/Arg836/Arg858). Biochemical assays reported an IC_50_ of 5–5.5 µM with marked positive cooperativity [81]. The complete conservation of this bracket across coronaviruses suggests pan-coronavirus potential, providing rare, high-resolution structural guidance for non-nucleoside design and a mechanistic counterpoint to promiscuous binders such as Suramin. As a complementary avenue, interface-mimicking peptides derived from Nsp8 disrupt the RdRp–Nsp8 interaction, suppress RdRp activity in cell-based assays, and reduce viral loads and mortality in mice after intranasal dosing [90]. This nominates the RdRp–Nsp8 interface as a therapeutically tractable site despite its apparent flatness [91].

#### 2.2.5. PK/PD

Remdesivir’s PK is characterised by quick conversion to active triphosphate in cells and a long intracellular half-life of the active form (~40 h) [92]. It must be administered IV once daily for five days; attempts at inhaled delivery are under exploration (to concentrate it in the lungs) [93]. Molnupiravir achieves high oral bioavailability, rapidly converting to N^4^-hydroxycytidine in plasma; it penetrates tissues including the lungs [94,95]. It is administered at a dose of 800 mg twice daily orally for five days. Molnupiravir’s active nucleoside has a short plasma half-life but is sufficient for incorporation into the replicating virus. Both drugs are well tolerated. However, Remdesivir can cause infusion reactions and liver enzyme elevations, and Molnupiravir can cause mild side effects such as headache or gastrointestinal (GI) upset [92,94]. Importantly, because efficacy hinges on very early treatment, outpatient-deployable formulations are critical, hence the emphasis on orally bioavailable nucleoside (pro)drugs such as Molnupiravir and Bemnifosbuvir (AT-527).

#### 2.2.6. Resistance

SARS-CoV-2′s RdRp is conserved across variants, but variants resistant to Remdesivir have been selected in vitro (e.g., the E802D mutation in RdRp conferring around 6-fold resistance) [96]. To date, clinical sequencing has not shown widespread polymerase mutations under drug pressure. Molnupiravir, by inducing mutations, could theoretically accelerate the emergence of new variants if some viruses survive suboptimal dosing (this is a debated issue) [97]. As of 2026, no specific SARS-CoV-2 mutations are universally accepted as bona fide Molnupiravir-resistance substitutions, although several putative RdRp candidates have been described [98,99]. The ExoN domain of Nsp14 is a resistance factor in general, as it can proofread and remove certain analogues (*vide infra*). Remdesivir partly evades proofreading owing to its structural mimicry of normal nucleotides after incorporation [77]. Therefore, strategies that combine a nucleoside with an ExoN inhibitor could prevent the virus from fixing mistakes, amplifying the nucleoside’s effect.

#### 2.2.7. Development Status

Remdesivir has been fully approved (use in hospitalised patients and as a 3-day outpatient IV course for high-risk early disease). Molnupiravir remains under emergency/conditional authorisation in several countries, although its uptake varies. Bemnifosbuvir (AT-527) reached Phase 2 for COVID-19 but failed to meet the primary endpoints, and its development has been de-prioritised. Deuremidevir (VV116; an oral prodrug of GS-441524) showed non-inferiority to Nirmatrelvir–Ritonavir in a randomised trial of mild-to-moderate COVID-19 [100]. China has also approved Azvudine (FNC), a repurposed nucleoside originally developed for HIV, for COVID-19 treatment [100,101]. To date, no non-nucleoside RdRp inhibitors have advanced into clinical trials [102].

#### 2.2.8. Nsp7 and Nsp8 Cofactors

Although not traditionally considered drug targets on their own, Nsp7 and Nsp8 have intriguing aspects. A study in 2023 identified a small pocket on Nsp7 that binds gallic acid and related small molecules [103]. Using NMR, researchers found gallic acid binds to a hydrophobic pocket on Nsp7 (not at the Nsp7–Nsp8 interface but on Nsp7’s surface). Although gallic acid is a very weak binder, this pocket could potentially be exploited to interfere with the assembly of the polymerase complex. Another approach has been the use of biologics: a nanobody (single-domain antibody) was developed targeting Nsp9 (an RNA-binding protein, but related to the replication complex), delivered via mRNA in lipid nanoparticles, which blocked viral replication in cells [104]. This strategy, essentially intracellular immunotherapy, demonstrates the creative tactics being considered to disrupt the RTC. It is conceivable that similar nanobodies could target the Nsp7–Nsp8 interfaces.

#### 2.2.9. Challenges and Opportunities

RdRp is a conserved machinery across all RNA viruses, and learning from decades of HIV/HCV polymerase inhibitor development is useful. One challenge is the proofreading ability of coronaviruses, which is almost unique among RNA viruses and reduces the efficacy of classic chain terminators [105]. However, this can be overcome by using analogues that trick the exonuclease or by co-inhibiting ExoN (*vide infra*). Another challenge is the design of allosteric inhibitors for such a large complex, and a deep understanding of transient pockets (via molecular dynamics simulations or fragment screening) may be needed. Opportunities include broad-spectrum nucleosides: some analogues (e.g., β-D-N^4^-hydroxycytidine from Molnupiravir) inhibit not only SARS-CoV-2 but also a range of RNA viruses, making them valuable for pandemic preparedness in general [106]. Polymerase inhibitors can also be made variant-proof by targeting absolutely conserved regions (e.g., the active site). The RdRp active-site motifs (A–G) are highly conserved; RdRp is ~96% identical between SARS-CoV and SARS-CoV-2 and the catalytic residues are invariant, helping explain why nucleotide analogues such as Remdesivir retain activity across variants and inhibit related β-coronaviruses (e.g., SARS-CoV and MERS-CoV) in vitro (notwithstanding rare resistance substitutions like Nsp12 E802D) [96,107,108]. This bodes well for the longevity of polymerase-based antivirals. In conclusion, RdRp in complex with Nsp7 and Nsp8 is a validated drug target. The success of Remdesivir and Molnupiravir underscores the potential of nucleoside analogues. Continued innovation aims to create orally bioavailable potent polymerase inhibitors that can be used alone or in combination with M^pro^ inhibitors to achieve synergistic suppression of SARS-CoV-2.

### 2.3. Clinical Benchmark: Replication Targets (M^pro^ and RdRp)

The programmes that have advanced furthest clinically (and the only ones with regulatory approvals to date) target the two replication “workhorses” M^pro^ and RdRp. This concentration reflects the convergence of factors, including clear catalytic mechanisms and high-quality structural data, robust industry-standard assay cascades, and medicinal chemistry precedents (peptidomimetic cysteine protease inhibitors and nucleos(t)ide analogues). Therefore, Table 1 serves as a field benchmark, summarising the most clinically validated chemical matter, setting exposure/PK, and resistance expectations for next-generation agents.

More than six years after the pandemic began, what does the therapeutic landscape look like? RdRp is validated by the nucleotide analogue Remdesivir (IV, hospital use) and the mutagenic ribonucleoside Molnupiravir (oral, outpatient); M^pro^ is validated by the oral protease inhibitor Nirmatrelvir co-dosed with Ritonavir, with additional regional approvals for other non-covalent M^pro^ inhibitors (e.g., Ensitrelvir). Together, these agents demonstrate that early, high target coverage can reduce progression risk, while also highlighting practical constraints: route of administration (Remdesivir), boosting/drug–drug interaction (DDI) (Nirmatrelvir/Ritonavir), and mechanism-linked safety and resistance considerations (Molnupiravir error catastrophe; emergent M^pro^ resistance pathways). By contrast, other RTC enzymes (*vide infra*: PL^pro^/Mac1, Nsp13, Nsp14, Nsp15, and Nsp16) remain preclinical or early translational. Table 1 anchors the manuscript’s prioritisation logic: build on the proven M^pro^/RdRp backbone and bring a second, orthogonal enzyme inhibitor toward the clinic to raise the barrier to escape (*vide infra*, see Section 5.2 and Section 5.3).

## 3. Near-Term Translational Priorities

The next wave of SARS-CoV-2 Nsp targets comprises those that have moved beyond conceptual interest and now show meaningful translational momentum but have not yet reached the level of clinical validation established for M^pro^ and RdRp. These targets combine a strong biological rationale with increasing structural and chemical tractability, and in several cases, are supported by emerging cellular or in vivo evidence that places them within the realistic reach of preclinical advancement. Importantly, they also address functions complementary to first-generation antiviral mechanisms, including polyprotein processing outside M^pro^, RNA proofreading, RNA capping, helicase-driven nucleic acid remodelling, and immune-evasion-linked processing of viral RNA byproducts. As such, PL^pro^, Nsp14, Nsp16, Nsp13, and Nsp15 represent the most credible near-term opportunities to expand the current antiviral arsenal beyond protease- and polymerase-centred therapy, particularly in the context of resistance-aware and mechanistically orthogonal combination therapy.

### 3.1. Papain-like Protease (PL^pro^, Belonging to Nsp3)

#### 3.1.1. Biology and Rationale

Nsp3 is a large, multi-domain protein (~200 kDa), the largest protein encoded by the coronavirus genome, with at least 15 domains described in SARS-CoV-2 [109]. Two notable enzymatic domains of Nsp3 are the papain-like protease (PL^pro^) and the macrodomain (Mac1) (*vide infra*) [110,111]. PL^pro^ cleaves the N-terminal portion of the polyprotein at three sites to release Nsp1, Nsp2, and Nsp3 [112]. PL^pro^ also has deubiquitinating and deISGylating activity, meaning it can remove ubiquitin and ISG15 (an interferon-stimulated ubiquitin-like protein) from host proteins [113]. This helps the virus antagonise innate immunity. PL^pro^ is essential for efficient replication and immune evasion, making it a prime target for antivirals [111]. Notably, blocking PL^pro^ not only inhibits virus replication by preventing proper polyprotein processing, but it also blunts the virus’s ability to subvert host ubiquitin/ISG15 signalling [114].

#### 3.1.2. Assays and Structural Biology

PL^pro^ assays typically measure the cleavage of fluorogenic peptides corresponding to the Nsp2|Nsp3 junction or test deubiquitination activity on ubiquitin–luciferase substrates [115,116,117]. High-throughput screens (HTS) have been conducted for PL^pro^ [117,118]. Crystallography has yielded multiple structures of PL^pro^ alone and in complex with inhibitors or substrates, aiding in structure-based design [119,120,121].

#### 3.1.3. Chemical Matter

**Figure 4:**
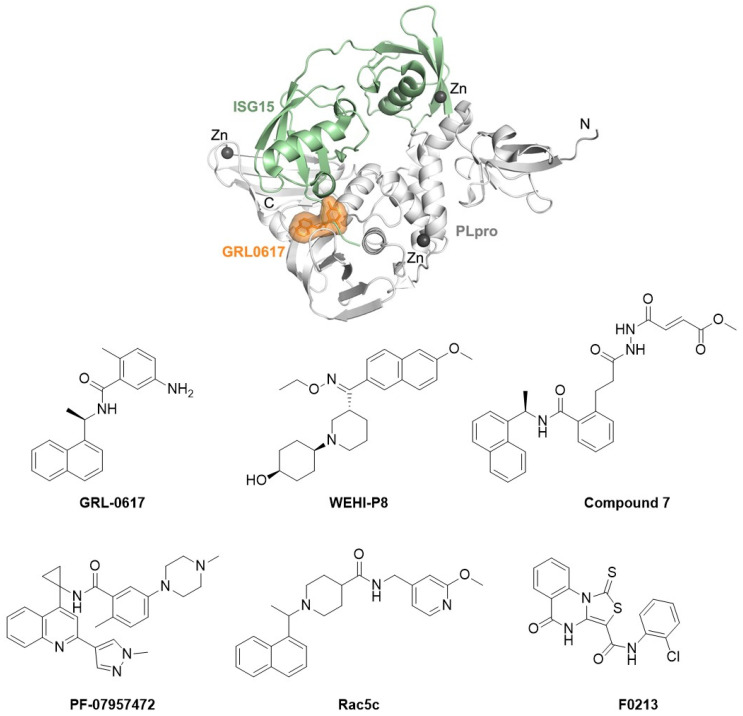
**Top:** Superposition of the crystal structure of the SARS-CoV-2 PL^pro^ C111S mutant in complex with ISG15 (PDB ID 6YVA) and the crystal structure of PL^pro^ bound to GRL0617 (PDB ID 7CJM). For clarity, only ISG15 from the 6YVA structure is displayed (green). PL^pro^ is shown in grey, GRL0617 in orange, and Zn ions in dark grey. **Bottom:** Representative chemotypes of SARS-CoV-2 PL^pro^, including GRL-0617 [122], PF-07957472 [123], Rac5c [124], F0213 [125], Compound 7 [126], and WEHI-P8 [127].

Early efforts were based on SARS-CoV PL^pro^ inhibitors. The non-covalent inhibitor GRL-0617, a naphthalene-benzamide (Figure 4), was initially developed against SARS-CoV and was found to inhibit SARS-CoV-2 PL^pro^ with an IC_50_ of 0.6 μM [122]. GRL-0617 binds to the S3/S4 pocket adjacent to the active site, preventing the enzyme from accommodating the distal ubiquitin/ISG15 motif [119]. Although GRL-0617 has good specificity (it does not hit human proteases), it showed relatively weak antiviral activity in cell culture (EC_50_ of 68 μM in Vero cells, even with P-gp efflux inhibited) [123]. This limited cell potency is thought to arise from poor cell permeability or efflux. Medicinal chemistry optimisation of GRL-0617 analogues was reported by Garnsey et al. (2024) and modifications on the naphthalene and aniline moieties led to improved potency [123]. One optimised PL^pro^ inhibitor from that series, PF-07957472, showed sub-μM antiviral efficacy and was efficacious in a SARS-CoV-2 mouse infection model, a milestone validating PL^pro^ as a druggable target. Rac5c is another early non-covalent PL^pro^ chemotype that engages the S3/S4 substrate-binding pocket, blocking PL^pro^’s deISGylase/deubiquitinase activity, inhibiting Nsp3 self-processing, and reducing SARS-CoV-2 replication in cells [124]. Although its potency and PK are modest, with no in vivo data reported, Rac5c serves as a useful tool compound to benchmark assays and seed SAR around non-covalent PL^pro^ inhibition.

Another screening identified F0213 (IC_50_ = 7.4 μM), a non-covalent inhibitor with broad coronavirus activity [125]. F0213 blocks both the proteolytic and deubiquitinating functions of PL^pro^, and it was suggested to allosterically interfere with the enzyme, possibly by binding near Lys157. Covalent inhibitors (Compound **7**) have also been explored, targeting the catalytic Cys111, but selectivity is a concern given the host deubiquitinases with similar active sites [126]. Regrettably, the most potent inhibitors in this series were deemed unsuitable for in vivo studies.

Finally, the WEHI-P series was recently reported [127]. Unlike inhibitors from the GRL-0617 family, these compounds engage a distinct binding pocket on PL^pro^ by inducing a conformational change in Met208. The lead compound, WEHI-P8 (IC_50_ = 12 nM, EC_50_ = 0.36 μM), demonstrated potent antiviral activity (potentially a pan-coronavirus antiviral) and was highly efficacious in a mouse model of severe acute disease, outperforming a Paxlovid-like treatment in reducing viral load and lung inflammation. Crucially, in a preclinical model that recapitulates long COVID-19 symptoms, early treatment with WEHI-P8 prevented long-term lung pathology and cognitive dysfunction, a protective effect that was not observed with the Paxlovid-like regimen. This study provides key proof-of-principle that targeting PL^pro^ may not only treat acute infection but also prevent the development of post-acute sequelae.

Overall, the PL^pro^ inhibitor landscape now comprises several mechanistically distinct chemotype classes. These include GRL-0617-derived non-covalent S3/S4 pocket inhibitors, including PF-07957472; alternative non-covalent scaffolds such as Rac5c and F0213; cysteine-directed covalent or reversible-covalent inhibitors such as Compound **7**, which target Cys111 but require careful selectivity profiling against host deubiquitinases; and crystallographic fragment hits that provide ligand-efficient starting points for structure-guided optimisation. Among these, PF-07957472 and WEHI-P8 currently provide the strongest evidence for progression beyond tool-compound status because they combine potent enzyme inhibition with cellular and in vivo antiviral activity.

#### 3.1.4. PK/PD and Safety

The PL^pro^ inhibitors reported (GRL-0617 analogues, F0213) are at the research stage and no detailed PK data have been reported. GRL-0617 has a moderate molecular weight of ~350 Da and a polar surface, suggesting potential cell permeability issues. New analogues with less efflux liability are being sought (Compound **7**) [126]. In terms of safety, PL^pro^ is virus-specific and, therefore, on-target toxicity is expected to be low. Off-target risks include human deubiquitinases; however, GRL-0617 is relatively selective [126]. One consideration is that PL^pro^ inhibitors might synergise with innate immune stimulation because PL^pro^ blockade restores ISG15 and ubiquitin signalling [114]. This could be beneficial but could also cause inflammation, and suitable in vivo models are needed.

#### 3.1.5. Development Status

Several academic groups and biotech firms are actively developing PL^pro^ inhibitors. A few patent applications have been filed for PL^pro^-targeting antivirals [128]. As of 2026, none have entered clinical trials. However, PF-07957472, Compound **7**, and WEHI-P8 are promising preclinical candidates. Dual inhibitors of PL^pro^ and other targets (or multi-target drug cocktails) may be a strategy for clinical use in the future.

#### 3.1.6. Challenges and Opportunities

PL^pro^ has a flexible active site because it must bind diverse substrates (polyprotein and ubiquitin/ISG15) [124]. Although this flexibility can complicate inhibitor design, it also creates opportunities to exploit multiple binding pockets, including both orthosteric and allosteric sites. One challenge is that coronaviruses can tolerate some changes in the PL^pro^ sequence, although the inhibitor-binding residues tend to be conserved across SARS-CoV and SARS-CoV-2 [129]. Surveillance for resistance mutations, such as those affecting the inhibitor–binding pocket, will be needed once PL^pro^ inhibitors are used. Moreover, combining a PL^pro^ inhibitor (which acts early, processing the N-terminus of the polyprotein) with an M^pro^ inhibitor (processing the rest of the polyprotein) might yield a one-two punch, preventing viral polyprotein maturation. Indeed, a recent study showed that a dual inhibitor targeting both PL^pro^ and M^pro^ active sites is feasible [130].

### 3.2. Nsp14: Proofreading and RNA Capping Functions

#### 3.2.1. Biology and Rationale

Nsp14 is a bifunctional enzyme with two domains: an N-terminal 3′–5′ exoribonuclease (ExoN) and a C-terminal guanine-N7 methyltransferase (N7-MTase). Nsp10 acts as an essential cofactor that binds Nsp14 to activate its ExoN activity (and similarly binds and stimulates Nsp16, *vide infra*) [131]. The ExoN domain of Nsp14 confers the ability to proofread RNA. It excises misincorporated nucleotides from the nascent RNA, thereby significantly increasing replication fidelity [132]. This proofreading function is why coronaviruses can have much larger genomes (~30 kb) than other RNA viruses, as they maintain lower mutation rates [66]. From a drug perspective, this makes Nsp14 ExoN an attractive target: its inhibition could synergise with nucleotide analogue drugs (which depend on viral error rates) [105]. The N7-MTase function of Nsp14 is involved in capping the viral mRNA (adding a methyl group to the 5′ cap guanosine), an essential step for mRNA stability, thus helping the virus evade innate immunity. Therefore, both activities of Nsp14 are essential for a viable virus, making Nsp14 a high-potential target.

#### 3.2.2. Assays and Structural Biology

ExoN activity is commonly measured using fluorescently labelled or radiolabelled RNA substrates, often bearing a mismatched 3′ terminus to mimic proofreading substrates. In complex with Nsp10, Nsp14 mediates 3′→5′ exonucleolytic cleavage, which can be detected by monitoring the loss of the 3′ terminal nucleotide(s) or the formation of shorter RNA products [133,134]. Several structures of Nsp14 have been determined. An early low-resolution SARS-CoV Nsp14–Nsp10 complex structure (PDB ID 5C8S) shows the arrangement of the ExoN active site (with two metal ions) and the MTase domain [135]. Recently, a cryo-EM structure of the SARS-CoV-2 Nsp14–Nsp10 complex was determined [136]. Structures of the N7-MTase domain with cap analogues and inhibitors have also been reported [137]. These structures illuminate drug–binding pockets: the ExoN active site binds metal ions and RNA, and the N7-MTase domain has a pocket for the methyl donor S-adenosylmethionine (SAM) and the guanine base. A 1.6 Å structure and biophysical characterisation of SARS-CoV-2 Nsp10, the highly conserved stimulator of Nsp14 ExoN, clarify its central role in viral RNA capping and provide a rigorous framework for designing Nsp10-targeted protein–protein interaction (PPI) inhibitors to disrupt viral replication [138].

Nsp10–Nsp14 assembly is dynamic: biophysical assays show that full-length Nsp10 binds Nsp14 with sub-μM affinity (K_d_ = 0.5 μM), whereas truncations (especially loss of the C-terminus) weaken binding by >20-fold, indicating that both the N- and C-terminal regions of Nsp10 contribute to optimal complex formation [139]. Hydrogen–deuterium exchange mass spectrometry (HDX-MS) further reveals transient contacts with the N7-MTase domain, implying intermediate association/dissociation states not captured by crystal structures and highlighting PPI-targetable surfaces beyond the canonical ExoN interface [139].

#### 3.2.3. Chemical Matter—ExoN Inhibitors

**Figure 5:**
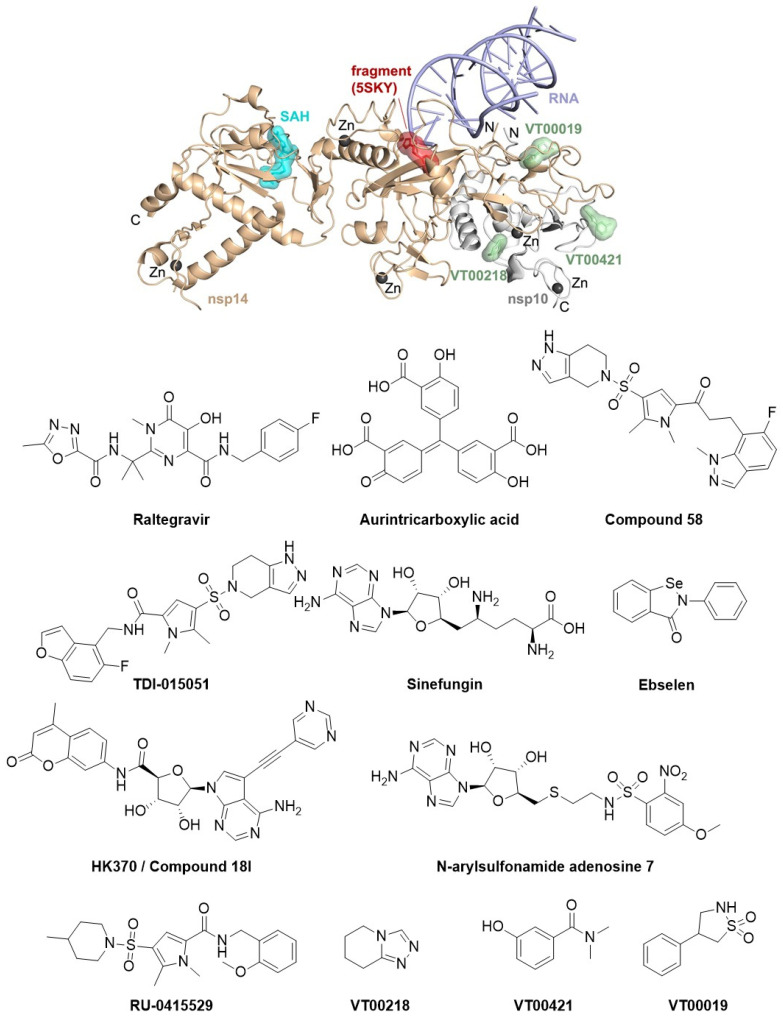
**Top:** Structure of the SARS-CoV-2 Nsp14–Nsp10–RNA complex (PDB ID 7N0B), with ligands superposed at the Nsp14–Nsp10 interface (green; PDB IDs 9FWH, 9FWQ, and 9FWU), the ExoN active site (red; PDB ID 5SKY), and the N7-MTase active site (cyan; PDB ID 7R2V). Nsp14 is shown in beige, Nsp10 in grey, RNA in purple, and Zn ions in dark grey. Ligand structures are shown in the top right (VT00019, VT00218, and VT00421). **Bottom:** Nsp14/Nsp10 chemical matter and structural context. Representative ExoN-directed inhibitors include aurintricarboxylic acid [141], Ebselen and Raltegravir [144], while representative N7-MTase ligands include the SAM mimic Sinefungin and HK370/compound 18L [146], N-arylsulfonamide adenosine 7 [147], RU-0415529 and TDI-015051 [148], and the optimised compound 58 [149]. Nsp10–Nsp14 interface fragments VT00019, VT00218, and VT00421 are also shown [151].

Because the ExoN function underpins coronavirus replication fidelity, its inhibition is expected to raise the viral mutation rate and, in principle, promote lethal mutagenesis, especially in combination with mutagenic nucleoside analogues [140]. Before COVID-19, there were essentially no selective small-molecule ExoN inhibitors. Since 2020, several tool compounds and screening-derived leads have emerged, although none yet qualify as drug-like ExoN-selective chemotypes. For instance, aurintricarboxylic acid (ATA) (Figure 5), reported since SARS-CoV-1 to inhibit ExoN, is a promiscuous polyanion that likely chelates catalytic metal ions and/or binds RNA non-specifically [141]. Recently, ATA analogues have been developed as ExoN inhibitors [142]. In addition, X-ray fragment screening of Nsp14 revealed dozens of hits across the ExoN and N7-MTase domains, including pockets at the Nsp10–Nsp14 interface and the Nsp14 hinge domain, providing concrete starting points for fragment-based drug discovery (FBDD)/allosteric design [143]. Finally, using a robust gel-based assay, Baddock et al. showed that the Nsp14–Nsp10 proofreading complex, although canonically a 3′→5′ exoribonuclease, can display broader RNase activity in vitro, including cleavage patterns consistent with internal incisions on certain RNA substrates. Using the same assay platform, they identified drug and drug-like inhibitors of Nsp14–Nsp10 activity, including Ebselen (better known as an M^pro^ inhibitor) and Raltegravir, thereby providing useful small-molecule starting points for ExoN-focused discovery campaigns [144]. These authors also found that ExoN activity is stimulated by the RdRp complex via Nsp8, reinforcing the value of orthogonal readouts to confirm on-target inhibition within the RTC. A focused review of this topic emphasised that ExoN proofreading blunts many nucleoside analogues, motivating combination strategies in which ExoN inhibition sensitises RdRp-targeting NAs [145].

#### 3.2.4. Chemical Matter—N7 MTase Inhibitors

The Nsp14 N7-MTase catalyses the transfer of a methyl group from SAM to the 5′ guanine cap of viral mRNA, forming a cap-0 structure. Inhibitors can either mimic the substrate (guanine analogues) or the cofactor SAM or occupy an allosteric pocket [137]. Early tools such as Sinefungin (a SAM analogue, IC_50_ = 0.46 μM) (Figure 5) established assay feasibility but lack selectivity over host MTases [146]. Building on SAM-mimetics, bisubstrate adenosine analogues that bridge the SAM and cap-binding sites (e.g., N-arylsulfonamide series) achieved sub-μM Nsp14 inhibition (e.g., N-arylsulfonamide adenosine 7, IC_50_ = 0.9 μM) and defined productive vectors for linker and aryl tuning [147]. Recent adenosine-5′-carboxamide derivatives, including coumarin analogues, refined this concept. HK370 (also reported as 18L) showed high selectivity, a favourable in vitro ADME/PK profile, and moderate cellular antiviral activity, providing an optimisable, enzyme-validated scaffold [146]. Parallel structure-based docking and ultra-large library campaigns delivered non-SAM chemotypes with biochemical activity and cellular signals, thus moving away from nucleoside-like properties [137].

A major advance is the discovery of the first non-covalent, non-SAM Nsp14 N7-MTase inhibitor series from high-throughput screening. Optimisation of RU-0415529 yielded TDI-015051, which binds in a ternary complex with Nsp14 and S-adenosyl-L-homocysteine (SAH), the demethylated product generated from S-adenosyl-L-methionine (SAM) after methyl transfer. This inhibitor is non-competitive with both SAM and GpppA, binds with a K_d_ of 61 pM, shows an EC_50_ value of 11 nM in cells, and demonstrates in vivo efficacy in mouse infection models comparable to Nirmatrelvir [148]. Follow-up medicinal chemistry details the SAR that enabled sub-nM (e.g., Compound **58**, IC_50_ = 0.25 nM) cellular potency and crystallographic confirmation of the ternary binding mode, establishing pharmacological validation of viral cap MTases [149]. TDI-015051 series properties are consistent with oral developability (improved permeability/solubility and sustained cellular potency), aligning PD with the ternary SAH–inhibitor mechanism that should translate across coronaviruses where the MTase site is conserved [149]. As an orthogonal approach, Bi(III) complexes allosterically inhibit both Nsp14 MTase and ExoN within the Nsp14/Nsp10 complex by displacing Zn(II) and inducing quaternary-structure rearrangements. They show antiviral activity in cells and animal models, making them useful mechanistic probes with multi-target potential, albeit outside classic small-molecule space [150]. Finally, for SAM-mimetic scaffolds (e.g., adenosine N-arylsulfonamides and 5′-carboxamides), enzyme potency is established, but cellular activity and selectivity remain the gating liabilities to optimise for systemic exposure.

#### 3.2.5. Chemical Matter—Nsp10-Nsp14 PPI Disruption

High-resolution crystal structures of the SARS-CoV-2 Nsp10–Nsp14 ExoN complex revealed the catalytic His268 in both active and inactive rotamers at 1.3 Å. An X-ray fragment screen (148 fragments) revealed five previously unreported ligandable pockets spanning the Nsp10–Nsp14 interface, the ExoN–hinge, and allosteric sites on Nsp10 (e.g., VT00019, VT00218, or VT00421) [151]. Nine related fragments clustered in a shared interface pocket, with the best hits binding at sub-mM K_d_ (0.51–0.56 mM), highlighting PPI disruption and hinge allostery as viable routes and providing merge/grow vectors for FBDD against ExoN activation. Additionally, X-ray fragment screening of Nsp10 identified four fragments in two conserved pockets (one overlapping the Nsp10–Nsp14 interface and the other at the Nsp10–Nsp16 interface), establishing ligandable sites for PPI disruption [152]. Microscale thermophoresis (MST) quantified a weak Nsp10–Nsp14 affinity (K_d_ 0.9–1.4 mM across ExoN and full-length constructs), consistent with a chemically dissociable complex.

#### 3.2.6. PK/PD and Safety

The lead non-SAM series (TDI-015051) couples pM target engagement with nM cellular potency and in vivo efficacy in mice (benchmarking near Nirmatrelvir), supporting drug-like exposure and on-target pharmacodynamics for N7-MTase inhibition. Its ternary SAH–inhibitor binding mode exploits Nsp14-specific features of the cap pocket, providing a structural rationale to minimise cross-inhibition of human cap MTases observed with SAM-competitive scaffolds (e.g., adenosine analogues) [148,149]. By contrast, ExoN programmes remain preclinical: tool compounds and metals can impair ExoN within the Nsp14–Nsp10 complex, but drug-like orthosteric inhibitors with clear cellular PD have not yet been established, underscoring the need for selectivity panels against host nucleases and RTC-aware assays linking ExoN block to nucleotide analogue sensitisation [144,150].

#### 3.2.7. Development Status

The most advanced programme is the Nsp14 N7-MTase series culminating in TDI-015051, a robust preclinical lead, but not yet in human studies. Follow-up medicinal chemistry confirmed the ternary SAH–inhibitor binding mode and improved properties consistent with oral developability, positioning the series for potential IND-enabling work. In contrast, ExoN efforts remain at the hit discovery/FBDD or tool-compound stage, and Nsp10–Nsp14 interface ligands are fragment-level with mM- to μM-range biophysical engagement, encouraging tractability.

#### 3.2.8. Challenges and Opportunities

Proofreading by the Nsp14–Nsp10 complex lowers coronavirus mutation rates and underpins the large genome size. ExoN-defective viruses show elevated error rates and attenuated fitness in cell and animal models, supporting ExoN as a potential drug target. Because this domain excises multiple nucleoside analogues, inhibiting ExoN is expected to sensitise RdRp-targeting NAs. ExoN-loss backgrounds increase susceptibility to agents such as Remdesivir or Ribavirin, motivating combination designs and RTC-aware PD assays. Selectivity remains central: orthogonal panels should de-risk activity against host nucleases and DNA-polymerase proofreading domains, while fragment maps and interface pockets provide allosteric/PPI-disruption routes beyond the metal/RNA orthosteric site [151]. Tool inhibitors (e.g., Ebselen, Raltegravir) demonstrate tractability but require translation into drug-like chemotypes with clear cellular PD [144].

Non-SAM, non-covalent Nsp14 N7-MTase inhibitors, such as TDI-015051, exhibit nM cellular potency and in vivo efficacy, validating the target. Resistance mutations that reduce susceptibility arise with fitness costs, and synergy with Nirmatrelvir supports combination backbones [148]. SAM-competitive series remain enzyme-potent but must improve selectivity versus human cap MTases. The ternary SAH–inhibitor binding mode exploited by TDI-015051 provides a structural pathway for selectivity [148].

Nsp10 cofactor and PPIs: Sub-mM Nsp10–Nsp14 affinity and fragment-defined pockets at the interface and hinge indicate PPI disruption as a complementary strategy; the dynamic assembly argues for full-length constructs and solution biophysics in screening to avoid false negatives [153].

Coronavirus mRNA capping relies on the Nsp16 (2′-O-MTase) and N7-MTase, both of which are stimulated by Nsp10. A survey of >13 million SARS-CoV-2 genomes highlighted that Nsp10 is highly conserved, with T12I, T102I, and A104V being the most frequent variants [154]. Structural and biophysical characterisation indicated that these substitutions have minimal impact on Nsp10–Nsp14/Nsp16 binding and stability, suggesting limited mutational escape capacity and supporting Nsp10-centred PPI disruption as a resilient therapeutic strategy. In summary, Nsp14 in complex with Nsp10 is a high-value target because of its proofreading and capping functions. Recent breakthroughs in the discovery of inhibitors for both of its activities have marked Nsp14 as one of the most exciting antiviral targets. Continued development could yield drugs that not only inhibit SARS-CoV-2 but also render it vulnerable to its own errors or immune detection, a one-two punch strategy for antiviral therapy.

### 3.3. Nsp16: 2′-O-Methyltransferase

#### 3.3.1. Biology and Rationale

Nsp16, in complex with Nsp10, is a 2′-O-MTase responsible for methylating the ribose 2′-OH of the first nucleotide of the viral mRNA cap, leading to cap-1 formation [155]. This step is crucial for disguising viral mRNA as host mRNA, as cap-1 formation helps evade innate immune sensors such as MDA5 and IFIT proteins [156]. Without Nsp16 activity, the virus’s mRNA would be flagged as “non-self” and trigger an immune response. Thus, Nsp16 is important for immune evasion and virulence. Inhibition of Nsp16 could make the virus more susceptible to host defences and directly reduce its replication efficiency. Humans have a 2′-O-MTase, named CMTR1, but it acts on host mRNA in the nucleus [157]. SARS-CoV-2 Nsp16 acts on viral mRNA in the cytosol, and structural differences exist to allow the development of specific inhibitors [158].

#### 3.3.2. Assays and Structural Biology

Nsp16 requires complex formation with Nsp10 for activity, as Nsp10 binding stabilises the SAM cofactor pocket [158,159]. Numerous structures of the Nsp16–Nsp10 complex exist, including SARS-CoV-2 Nsp16–Nsp10 bound to a cap analogue and Sinefungin [160]. These reveal a well-formed active site pocket for SAM and a separate binding groove for capped RNA. High-throughput assays measure the transfer of a radiolabelled methyl group from ^3^H-SAM to a short, capped RNA substrate [161]. Alternatively, methylation can be detected using mass spectrometry. For screening, coupling assays (such as MTase-Glo that generates light when SAM is consumed) or fluorescence polarization (FP)-based RNA displacement assays can be used [162,163]. Additionally, a radiometric scintillation proximity assay (SPA) enabled screening cascades (virtual-to-biochemical-to-cellular) and triage by site occupancy, facilitating structure-guided optimisation [164].

#### 3.3.3. Chemical Matter

**Figure 6:**
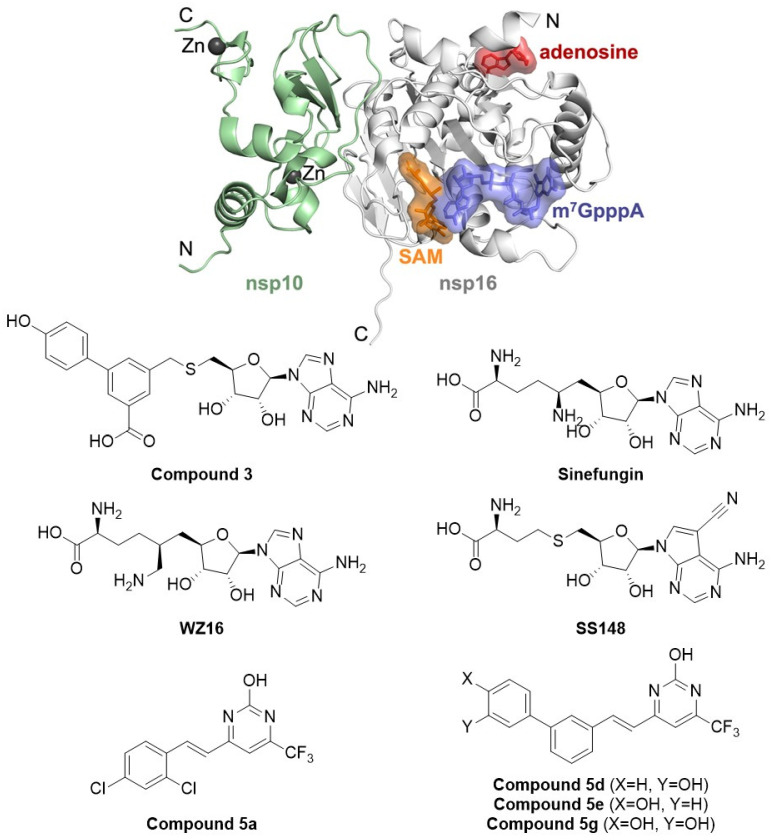
**Top:** Crystal structure of the SARS-CoV-2 Nsp16–Nsp10 complex with bound SAM (orange), the cap analogue m^7^GpppA (blue), and adenosine (red) (PDB ID 6WKS). Nsp16 is shown in grey, Nsp10 in green, and Zn ions in dark grey. **Bottom:** Representative Nsp16 inhibitor scaffolds, including the SAM mimic Sinefungin and substrate-based adenosine analogue Compound 3 [165], the pan-coronavirus SAM-site probes SS148 and WZ16 [167], and the allosteric covalent pyrimidin-2-ol series represented by Compound 5a and the optimised 5d/5e/5g derivatives [166].

Early SAM-competitive tools, such as Sinefungin (Figure 6), established tractability but lacked selectivity. Currently, co-crystal structures define a dual-site binding mode in which substrate-based adenosine analogues occupy the SAM subsite and project into the RNA (cap) groove of the target. Exemplars achieved low-nM inhibition of the Nsp16–Nsp10 complex (e.g., Compound **3** IC_50_ = 0.8 nM), with clear H-bonding and π-stacking vectors that map potency-driving interactions for SAR [165]. Orthogonally, Inniss et al. discovered a druggable cryptic pocket adjacent to the SAM cleft, yielding a covalent pyrimidin-2-ol series [166]. Lead 5a showed time-dependent inhibition with crystallographic confirmation of covalent engagement, and optimised analogues (5d/5e/5g) retained irreversible MTase inhibition and displayed cellular antiviral activity against Mouse Hepatitis Virus (MHV) in L2 cells (6–15 µM), albeit with cell-line-dependent cytotoxicity in some SARS-CoV-2 models. These data validate the allosteric inhibition of Nsp16 as a viable route beyond SAM competition. Complementing these advances, a recent SPA for Nsp16–Nsp10 enabled screening cascades (virtual-to-biochemical-to-cellular) that yielded cell-active hits and classified chemotypes by site occupancy, providing a practical engine for SAR and selectivity optimisation alongside structural analysis. Finally, broad-spectrum benchmarking across all seven human coronaviruses revealed that Nsp16 exhibits low-nM RNA K_m_ (20–140 nM) and low-µM SAM K_m_ values on a cap-1-like substrate [167]. SS148 and WZ16 inhibit the Nsp10–Nsp16 complex (SS148 IC_50_ = 0.2–1.5 µM; WZ16 = 2.5–24 µM, species/substrate dependent), underscoring conserved pharmacology while providing useful starting points for broader-spectrum optimisation, albeit with the caveat of modest potency [167].

#### 3.3.4. PK/PD and Safety

Dual-site adenosine analogues deliver enzyme-level low-nM potency with structural confirmation. The translational risk remains selectivity versus human cap MTases, which the dual-site engagement and shape complementarity to the viral RNA channel could help mitigate, but must be demonstrated experimentally [165]. Pan-coronavirus profiling indicates cell-active SAM-site inhibitors SS148 and WZ16 with µM potency. These serve as breadth probes but highlight the need to optimise permeability, cellular potency, and human-MTase selectivity for drug-like exposure [167]. For allosteric chemotypes, cell-line-dependent cytotoxicity and time-dependent covalency require careful PD interpretation and off-target de-risking. Nonetheless, the cryptic pocket provides a structurally validated path to non-SAM inhibition with cellular signals in coronavirus models [166]. SPA-based workflows support PK/PD linkage by enabling site-mechanism classification of hits prior to cellular assays [164].

#### 3.3.5. Development Status

The most advanced assets remain preclinical. They include dual-site, substrate-based adenosine analogues with low-nM enzyme potency and clear structure-guided paths towards selectivity [165]; allosteric covalent tools targeting a cryptic pocket, with low-µM cellular activity in MHV and crystallographic validation [166]; and the pan-coronavirus SAM-site probes SS148 and WZ16, which show µM potency across human coronaviruses [164]. Finally, the standardisation of viral species panels, primary airway cell assays, and human MTase selectivity will be key milestones for hit-to-lead progression.

#### 3.3.6. Challenges and Opportunities

The SAM and cap-RNA subsites of Nsp16 are highly polar, and inhibitors designed to mimic SAM or the cap substrate often inherit similar polar physicochemical properties, creating challenges for membrane permeability and selectivity over human MTases. Structural studies have shown that dual-site scaffolds can engage the SAM pocket and extend into the RNA groove to gain potency and shape complementarity, thereby providing a structural basis for selectivity, but they still require careful optimisation for cell entry and oral drug-like properties [165]. In addition, the discovery of a cryptic/allosteric pocket adjacent to the SAM cleft enables non-SAM mechanisms with greater latitude in the physicochemical space. Covalent pyrimidin-2-ol tools validate this site and show cellular antiviral activity in coronavirus models, albeit with time-dependent inhibition and cell line-dependent cytotoxicity that must be engineered during lead optimisation [166]. However, because Nsp16 activity depends on Nsp10 and a capped viral RNA substrate, screening should use the Nsp10–Nsp16 complex together with RNA substrates that mimic the native 5′ cap context, and incorporate site-aware workflows to classify SAM-site versus allosteric binders before cellular triage.

Functional constraints on capping and the conserved architecture of Nsp16 across human coronaviruses support pan-coronavirus potential. Broad profiling shows shared inhibitor sensitivity across human coronavirus Nsp16s (e.g., SS148 and WZ16), while sequence differences highlight the need for cross-species SAR and resistance surveillance [164]. Inhibiting Nsp16 is expected to enhance innate sensing of viral RNA (cap-0/IFIT1 vulnerability). Allosteric inhibitors already show innate-sensitising phenotypes in cells, supporting combinations with polymerase inhibitors or immune modulators where safety allows. Synergy with Nsp14 N7-MTase inhibition (*vide supra*) is mechanistically attractive for reducing capping capacity on two fronts. Overall, the Nsp16–Nsp10 complex offers two complementary avenues, dual-site SAM/cap mimetics and allosteric cryptic-site binders, with clear structural guidance. The main opportunities lie in translating enzyme-level potency into selective, cell-active, and orally developable leads with broad spectrum activity against coronaviruses.

### 3.4. Nsp13: RNA Helicase

#### 3.4.1. Biology and Rationale

Nsp13 is a superfamily-1 (SF1) helicase and RNA 5′-triphosphatase that translocates 5′→3′ on nucleic acids, using NTP hydrolysis to unwind duplex RNA/DNA and initiate cap formation. It is essential for replication of the ~30 kb genome, associating with the RdRp complex, to resolve RNA secondary structures during synthesis. Nsp13 is highly conserved among closely related sarbecoviruses, including SARS-CoV and SARS-CoV-2, and preserves core helicase and ATPase motifs across β-coronaviruses, underscoring its strong functional constraint [168]. Beyond its unwinding role, Nsp13 antagonises innate immunity, most notably by binding TBK1 to blunt IRF3 activation and type-I interferon signalling. It also engages the host deubiquitinase USP13, which stabilises Nsp13 and further dampens interferon (IFN) responses [169]. Given its essential enzymatic function and limited homology to human SF1 helicases at drug-binding surfaces, Nsp13 is a potential antiviral target, albeit helicases have historically been difficult to drug.

#### 3.4.2. Assays and Structural Biology

Helicase activity is measured by assays in which it unwinds a labelled double-stranded oligonucleotide (one strand is often radiolabelled or fluorescently tagged). ATPase activity (NTP hydrolysis) can also be monitored via colorimetric or luminescent phosphate detection [170,171]. Several structures of Nsp13 exist, showing the arrangement of the five domains: zinc-binding, stalk, 1B, RecA1 and RecA2 [172]. Importantly, a cryo-EM structure places Nsp13 within the polymerase RdRp/Nsp7/Nsp8 complex, where two Nsp13 molecules associate with the RTC to help translocate along RNA [173]. These structural details reveal pockets at the ATP binding site and at the junction of the 1A/2A domains, offering potential drug-binding sites.

#### 3.4.3. Chemical Matter

**Figure 7:**
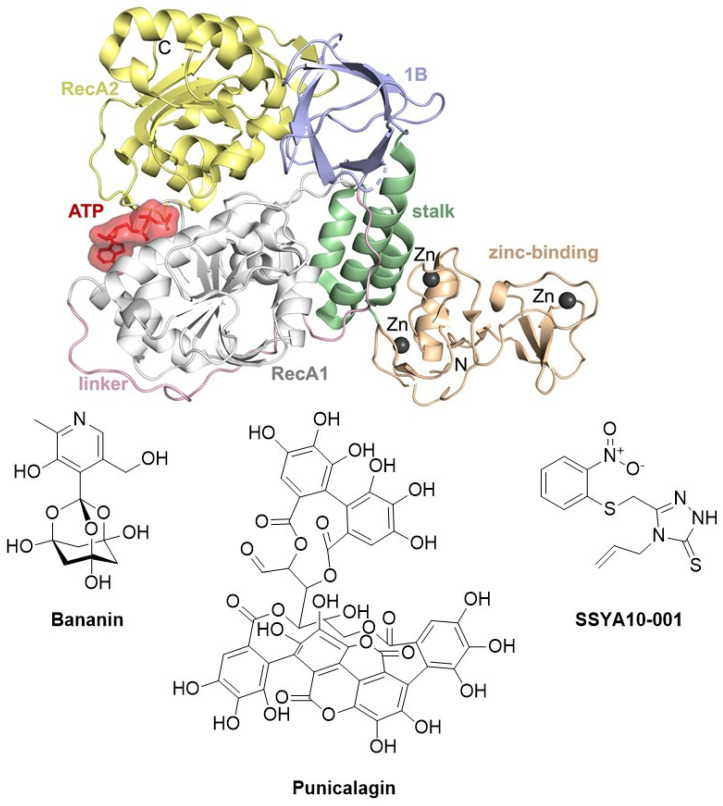
**Top:** Crystal structure of SARS-CoV-2 Nsp13 in complex with ATP (PDB ID 9I53). The zinc-binding domain is shown in beige, the stalk domain in green, the 1B domain in purple, the RecA1 domain in grey, the RecA2 domain in yellow, the linker between the 1B and RecA1 domains in pink, ATP in red, and Zn ions in dark grey. **Bottom:** Representative reported helicase inhibitors: Bananin [174], Punicalagin [177], and SSYA10-001 [177].

Early “broad-spectrum” helicase chemotypes such as Bananins (Figure 7) [174,175] and Suramin [176]. (*vide supra*, RdRp section) showed activity against SARS-CoV and SARS-CoV-2 helicase. However, their non-specific, multi-target behaviour and poor drug-like properties such as high charge and polarity, and frequent aggregation, confine them to tool-compound status rather than leads. Recent structural work mapped two druggable and highly conserved pockets on helicase and delivered 65 crystallographic fragment hits, providing concrete anchor points for structure-guided campaigns [172]. Complementing this, an AViDD-led HTS (∼650,000 molecules) built a robust 1536-well helicase assay (average Z′ ≈ 0.86) and identified 674 compounds with IC_50_ < 10 μM, establishing a sizeable, tractable starting set [177]. Specific small-molecule exemplars now include SSYA10-001, which inhibits SARS-CoV-2 helicase and retains activity against the prevalent R392C Omicron-lineage variant, and the natural product Punicalagin, an allosteric inhibitor that binds at the 1A/2A interface (K_d_ = 21.6 nM) and suppresses replication in cells (EC_50_ = 0.20–0.35 μM).

Despite sustained efforts, no helicase inhibitor has yet matched the potency of M^pro^ leads. The core hurdle is selectivity: the helicase catalytic site binds ATP, which is used by many host enzymes. Therefore, small molecules must exploit Nsp13-specific features, such as the RNA-binding channel, allosteric pockets between domains, or the conformational cycle that couples ATP hydrolysis to translocation. Several reported “selective” chemotypes likely work by stabilising an ADP-bound or “open state” that is incompetent for nucleic-acid engagement or product release. Encouragingly, recent high-resolution structures, fragment maps, and validated hit sets provide a solid foundation for FBDD/SBDD optimisation toward oral-drug-like scaffolds with improved on-target selectivity.

#### 3.4.4. PK/PD and Safety

No helicase-directed compounds have yet demonstrated in vivo efficacy, and their ADME and pharmacokinetic properties remain insufficiently characterised in human-relevant systems. Suramin illustrates the pitfalls of early chemotypes such as parenteral administration, high polarity, and broad off-target liabilities. Therefore, it serves as a tool rather than a lead. A clinically viable helicase inhibitor should achieve rapid, sufficient exposure in the airway epithelium early in infection, favouring oral or inhaled delivery with high lung:plasma ratios, show clean selectivity versus host helicases/NTPases, especially human superfamilies 1 and 2 RNA/DNA helicases and the mitochondrial replicative helicase TWNK (“Twinkle”), and avoid generic liabilities (P-gp efflux, lysosomal trapping, hERG liabilities, phospholipidosis). The moderate interspecies conservation of Nsp13 across β-coronaviruses (>70% identity between SARS-CoV-2 and MERS-CoV) suggests that a single well-tuned scaffold may not deliver pan-coronavirus breadth [178]. Even more important, antiviral selectivity must be demonstrated explicitly with host helicase and mitochondrial safety panels before first-in-animal studies [179].

#### 3.4.5. Development Status

Helicase inhibitors are still in the early stages of discovery. Due to its essential role and high degree of conservation, Nsp13 is considered a high-value target for pan-coronavirus therapeutics, despite the potential challenges in achieving universal breadth across all lineages. Nevertheless, inhibitors could also potentially act on other viral families with related helicases (though coronavirus helicases are somewhat unique to Nidoviruses) [173]. Given the difficulty, one strategy considered is targeting a host cofactor if any are needed by Nsp13. However, helicase largely works independently (with some stabilisation from Nsp8/Nsp12) [180]. Another strategy is to disrupt the interaction of Nsp13 with the RTC. For instance, helicase binds to Nsp8 and possibly RdRp, and a peptide or small molecule that interrupts that interface could indirectly inhibit helicase function. This is being explored by leveraging the structural data of helicase–RTC.

#### 3.4.6. Challenges and Opportunities

Few licensed antivirals directly target the viral helicases. While herpesvirus helicase–primase inhibitors (exemplified by Amenamevir) provide a precedent [181], no RNA-virus helicase inhibitor has been approved. Nsp13’s mechano-chemical cycle involves large conformational changes during ATP binding/hydrolysis and translocation, likely creating transient, allosteric pockets that can be exploited [182]. Advances such as time-resolved cryo-EM and crystallography, alongside fragment maps, should help reveal and stabilise these states for structure-enabled design. Potency and selectivity versus host helicases remain key hurdles, and with two Nsp13 copies per RTC, shallow inhibition may be buffered. Nevertheless, partial conformation-locking inhibitors can still sufficiently slow replication, especially in combination with RdRp or protease inhibitors, to tip the balance toward immune clearance.

In terms of opportunities, Nsp13 helicase inhibitors could be deployed in combination with other DAAs in future outbreak settings. Although the catalytic helicase core and ATPase motifs are strongly conserved, peripheral and allosteric regions may vary more substantially across distant coronaviruses. Therefore, a scaffold optimised only against SARS-CoV-2 may not automatically deliver pan-coronavirus breadth; early cross-species biochemical, structural, and cellular profiling will be required to prioritise inhibitors that engage conserved functional pockets. At the same time, the strong functional constraints on Nsp13 may increase the barrier to resistance, as mutations that reduce inhibitor binding could also impair helicase activity and viral fitness. Consistent with this, relatively few Nsp13 substitutions have been observed among major SARS-CoV-2 variants compared with more variable viral proteins, although this observation should be interpreted cautiously until clinically validated helicase inhibitors impose direct selective pressure [183]. In summary, Nsp13 is a tantalising but challenging target. Progress is being made in the discovery of hits. A concerted effort similar to that devoted to HCV helicase inhibitors could still yield a viable candidate, although none of those inhibitors has yet reached approval.

### 3.5. Nsp15: Uridylate-Specific Endoribonuclease (NendoU)

#### 3.5.1. Biology and Rationale

Nsp15 is a 345-amino-acid endoribonuclease that preferentially cleaves RNA 3′ of uridines, hence its designation as a poly(U)-specific endonuclease (NendoU) [184]. It is highly conserved across coronaviruses (e.g., 88% sequence identity between SARS-CoV and SARS-CoV-2) [185], underscoring its important role in the viral lifecycle. Unlike the exonuclease Nsp14, Nsp15 is not strictly required for viral RNA synthesis in cell culture [184]. Instead, its principal function is to help the virus evade host innate immunity. Nsp15 specifically degrades viral RNA byproducts (particularly polyuridine sequences in double-stranded RNA replication intermediates), which would otherwise trigger intracellular sensors and induce interferon responses. Coronaviruses with catalytically inactive Nsp15 replicate but are attenuated: for example, Nsp15-deficient mutant viruses in mice and pigs showed markedly reduced virulence, elevated interferon levels, and rapid clearance by the host [186]. By trimming 5′-polyU stretches on negative-sense RNAs, Nsp15 “hides” the virus from RNA sensors, making it a critical virulence factor. This immune evasion role provides a clear rationale for targeting Nsp15, and inhibiting its nuclease activity could unmask the virus to innate defences, thereby suppressing infection. Notably, because Nsp15 activity is not essential for genome replication *per se*, an Nsp15 inhibitor might not completely block virus growth in cell culture on its own, but it could synergistically reduce pathogenicity and replication fitness in vivo by allowing immune recognition [184]. The high conservation of Nsp15 and the severe fitness cost of Nsp15 loss reinforce its attractiveness as an antiviral target [185].

#### 3.5.2. Assays and Structural Biology

Nsp15 forms an active hexamer (dimer of trimers), and oligomerisation is essential for properly organising the catalytic site and conferring positive cooperativity [184]. Each 38–39 kDa protomer contains an N-terminal oligomerization domain (ND), a middle domain (MD), and a C-terminal endoribonuclease (NendoU) domain. The six active sites face the hexamer central pore. Catalysis occurs at the His235, His250, Lys290 triad and follows an RNase-A-like mechanism [185]; His250 activates the RNA 2′-OH for in-line attack, His235 protonates the leaving 5′-oxygen, and Lys290 stabilises the transition state, yielding a 2′,3′-cyclic phosphate and a 5′-OH [187]. Uridylate selectivity is mainly enforced by Ser294, Thr341, Tyr343, which shape a U-recognition pocket [185]. Nsp15 is only active as a hexamer. A truncated monomeric form (lacking the oligomerisation domain) was crystallised from SARS-CoV-1 and showed a distorted active site and loss of function, suggesting that protomer–protomer interactions serve as an allosteric switch to organize the active site properly [184]. Consistent with this, kinetic studies show the enzyme exhibits positive cooperativity within the hexamer. For instance, substrate binding and turnover are enhanced by the oligomeric assembly [188].

Biochemically, Nsp15 endonuclease activity is often measured using short RNA substrates containing uridine and detectable labels. A common high-throughput assay is a fluorescence resonance energy transfer (FRET) cleavage assay: for example, an RNA oligonucleotide labelled with a 5′ fluorophore (e.g., FAM) and 3′ quencher will yield increased fluorescence when Nsp15 cleaves it and separates the quencher [189]. In addition to FRET assays, radiolabelled RNA cleavage assays have been used. For instance, an internally ^32^P-labeled poly(U) RNA or a short 5′−^32^P RNA can be incubated with Nsp15 and the cleavage products can be separated by gel electrophoresis [190].

Structurally, Nsp15 has been studied using X-ray crystallography and cryo-EM, revealing insights into its dynamics. Cryo-EM studies of SARS-CoV-2 Nsp15 further revealed that the enzyme can adopt at least two distinct conformational states: an “open” state where the endoribonuclease domain is positioned to accommodate substrate, and a “closed” state in which active-site loops constrict, potentially representing an inactive or post-catalytic state [188]. pH-dependent movements and rotations of the endoU domain have been observed, hinting at an inherent flexibility that might regulate catalysis. This conformational malleability is of interest because it suggests additional allosteric mechanisms for the regulation of the protein.

#### 3.5.3. Chemical Matter

**Figure 8:**
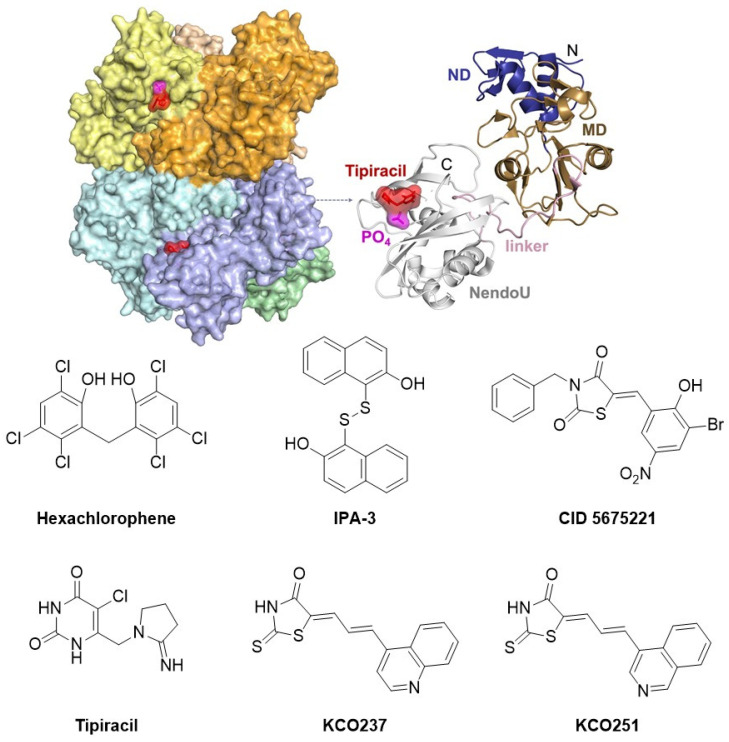
**Top left:** Hexameric structure of SARS-CoV-2 Nsp15 (PDB ID 6WXC). The six protomers are coloured orange, yellow, beige, purple, cyan, and green. Tipiracil and phosphate ions in the active sites are shown in red and magenta, respectively. **Top right:** Crystal structure of SARS-CoV-2 Nsp15 in complex with Tipiracil (red) and a phosphate ion (magenta) in the active site (PDB ID 6WXC). The ND is shown in dark blue, the MD in brown, the linker in pink, and the EndoU domain in grey. **Bottom:** Representative reported small-molecule hits, including Tipiracil [190], Hexachlorophene, IPA-3, and CID 5675221 [189], and the NendoU inhibitors KCO237 and KCO251 [86].

While Nsp15 remains an attractive therapeutic target, no specific antivirals have yet advanced to clinical development; nevertheless, various inhibitor chemotypes have been identified through repurposing and high-throughput screening. The most prominent active-site inhibitor is Tipiracil (Figure 8), an uracil analogue approved for use in oral chemotherapy (Trifluridine/Tipiracil). Tipiracil (IC_50_ = 5–15 µM) was identified early in the pandemic as a potential Nsp15 binder owing to its similarity to uridine [190]. Structural studies confirmed that Tipiracil inserts into the uridine-binding pocket of the Nsp15 active site (PDB ID 6WXC). In addition to Tipiracil, several high-throughput screens have identified Nsp15 inhibitors with diverse mechanisms. A recent screen of >100,000 compounds yielded five chemically distinct lead inhibitors [189], including hexachlorophene (a polyphenolic antiseptic), IPA-3 (a pyrimidinone known as a PAK1 kinase inhibitor), and a sulfonamide analogue (CID 5675221). These compounds inhibited SARS-CoV-2 replication in cell culture at sub-cytotoxic concentrations, showed low-μM potency in enzymatic assays, and achieved measurable antiviral effects in cells, making them valuable starting points for drug development. Notably, IPA-3 was found to act as an irreversible inhibitor of Nsp15, likely by covalently modifying one or more cysteine residues of this enzyme. A recent study reported that Thiazolidinedione and Rhodanine analogues are first-in-class NendoU inhibitors [86]. The lead compounds KCO237 (IC_50_ = 0.3 μM) and KCO251 (IC_50_ = 0.9 μM) suppressed SARS-CoV-2 replication in Vero E6 cells at non-toxic concentrations. Together, these chemotypes provide tractable starting points for structure-guided optimisation of selective Nsp15 inhibitors.

Looking ahead, the discovery of allosteric sites on Nsp15 opens the door for the development of non-active-site inhibitors. Fragments binding at the Nsp15 interdomain interfaces could, in principle, disrupt the hexamer or stabilise an inactive conformation. Such allosteric inhibitors might avoid the challenge of competing with RNA substrates in the highly polar active site. Indeed, a comprehensive fragment-based crystallographic screen (>1400 fragments) was recently performed by Godoy et al., identifying several small-molecule binding sites outside the active site [188]. This fragment screen identified several ligandable pockets on the Nsp15 surface. Future campaigns could exploit these to design larger drug-like, potent inhibitors. Any small molecules that prevent Nsp15 from assembling into its active hexamer or lock the enzyme in a “closed” state would effectively shut down its nuclease function. Although no advanced allosteric inhibitors have been reported, this strategy is an active area of research.

#### 3.5.4. Translational Considerations (Resistance and Opportunities)

Nsp15 remains an attractive yet challenging target for antiviral development. Its conservation across coronaviruses supports the possibility of broad-spectrum inhibition and aligns well with pandemic preparedness goals [184,189]. In principle, this conservation may also favour a relatively high barrier to resistance because mutations in or near the catalytic site can compromise enzymatic function and reduce viral fitness [191]. However, recent studies have shown that coronaviruses can acquire Nsp15 inhibitor escape mutations, even if these come with fitness costs [192]. Likewise, functional disruption of Nsp15 does not invariably cause severe attenuation but rather produces context-dependent defects that are most apparent in settings where innate immune control is relevant. Because Nsp15 contributes primarily to immune evasion and broader viral fitness rather than serving as a primary replication enzyme, its greatest value may lie as a complementary target in combination regimens [193,194]. Finally, although Nsp15 lacks a close human counterpart in the viral replication machinery, humans do encode an EndoU homolog and, therefore, selectivity over host nucleases remains an important medicinal chemistry consideration. Overall, Nsp15 is best viewed as a promising adjunctive target whose inhibition could strengthen current antiviral strategies while contributing to broader coronavirus preparedness and responses.

## 4. Emerging but Strategically Important Targets

A further group of Nsps remains at an earlier stage of chemical validation but retains strategic importance because of their distinctive biology, mechanistic complementarity, and potential relevance to long-term coronavirus preparedness. These targets are not yet supported by the same depth of translational evidence as the benchmark or near-term priority tiers, and in some cases, the available chemical matter remains limited, indirect, or predominantly tool compounds. Nevertheless, they continue to merit attention because they illuminate underexploited vulnerabilities in viral gene expression, host interaction control, and replication complex regulation, which may become increasingly actionable as assay systems, structural data, and screening approaches improve. Nsp1, the Nsp3 macrodomain (Mac1), and related emerging intervention nodes occupy an important exploratory space in the antiviral landscape. Although they are not immediate front-runners for clinical translation, they may help define the next generation of unconventional yet mechanistically valuable antiviral agents.

### 4.1. Nsp1: Host Translation Suppressor

#### 4.1.1. Biology and Rationale

Nsp1 is a 180 amino-acid protein that binds to the 40S ribosomal subunit and blocks host mRNA entry into the ribosome, effectively shutting down host protein synthesis [195]. By doing so, Nsp1 also dampens the host innate immune response (e.g., interferon production) [196,197]. The C-terminal helical region of Nsp1 (amino acids 150–180) inserts into the ribosomal mRNA channel, mimicking host translation initiation factors [195]. This unique mechanism makes Nsp1 an attractive target and disabling Nsp1 could restore host antiviral protein synthesis and tip the balance in favour of the host during infection [198]. In animal models and primary cells, Nsp1 is required for efficient replication and pathogenesis [195,199,200].

#### 4.1.2. Assays and Structural Biology

Structures of Nsp1 bound to the 40S ribosome via cryo-EM have confirmed the binding mode and key residues involved [201]. These structural insights enabled in silico drug and fragment-based screening against two functional regions of Nsp1: the RNA-binding groove within the N-terminal domain and the C-terminal helix–loop–helix region [202]. Cell-based assays for Nsp1 function often measure the restoration of reporter gene translation in the presence of Nsp1, or Nsp1’s inhibition of interferon-stimulated gene expression [203].

#### 4.1.3. Chemical Matter

**Figure 9:**
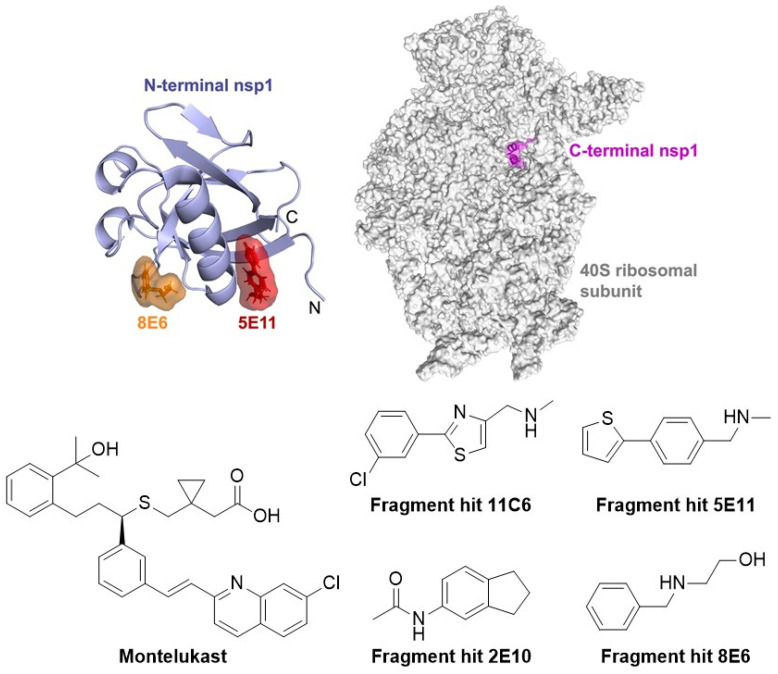
**Top left:** Crystal structure of the SARS-CoV-2 N-terminal Nsp1 domain (grey), with representative ligands superposed at binding site I (red; PDB ID 8CRF) and binding site II (orange; PDB ID 8AZ8). **Top right:** Structure of the 40S ribosomal subunit (grey) bound to the SARS-CoV-2 C-terminal Nsp1 region (magenta) (PDB ID 6ZLW). **Bottom:** Chemical starting points for Nsp1 inhibition, including Montelukast, a reported Nsp1 binder that rescues Nsp1-mediated translation shutoff [204], and fragment hits 11C6, 5E11, 8E6, and 2E10 [207].

Through virtual screening of FDA-approved drugs, Montelukast (an asthma drug) (Figure 9) emerged as a top hit binding to the Nsp1 C-terminus [204]. Montelukast was found to bind Nsp1 with a K_d_ of 10.8 μM in vitro and form a stable Nsp1–Montelukast complex in molecular dynamics simulations. In cells, Montelukast relieved Nsp1-mediated translation inhibition (rescuing a luciferase reporter) and reduced SARS-CoV-2 replication in the infected cell culture. These data validate small molecule targeting Nsp1’s ribosome-binding function and exhibit antiviral activity. Montelukast is considered a lead compound for Nsp1 and, potentially, analogues could improve its affinity. In line with this, Kao et al. identified synergistic repurposed combinations that blunt Nsp1 activity. A combination of Ponatinib, Rilpivirine, and Montelukast reversed Nsp1-driven translational shutdown to levels comparable with Nsp1 loss-of-function and mitigated Nsp1 toxicity in cells, supporting combination strategies for Nsp1 targeting [202]. These data reinforce Montelukast’s on-target contribution within multi-drug regimens and further validate the disruption of the Nsp1–ribosome interface as a druggable mechanism.

In addition, studies have identified cryptic pockets in the N-terminal Nsp1 domain via NMR and crystallography that accommodate small ligands (Figure 9) [205]. For example, fragment-based X-ray screening revealed two distinct ligand-binding sites on Nsp1 [206]. Furthermore, using anomalous diffraction at two low X-ray energies, Ma et al. showed that fragments containing sulphur or chlorine substituents (e.g., 11C6) bound to Nsp1 can be placed with high confidence, resolving multiple orientations and improving FBDD model accuracy, which is useful for refining SAR studies [207].

#### 4.1.4. PK/PD and Safety

As an approved asthma drug, Montelukast is orally available with a well-characterised human safety profile [208]. However, achieving sufficient concentrations at the site of SARS-CoV-2 replication (e.g., lung tissues) is necessary. Currently, no dedicated ADME studies have been reported for Nsp1 inhibitors but repurposed drugs such as Montelukast provide a head start on safety considerations.

#### 4.1.5. Development Status

Currently, there are no potent lead compounds for Nsp1. The proof-of-concept demonstrated by Afsar et al. in 2022 using Montelukast has spurred interest in Nsp1 [204]. For this reason, Montelukast was explored in some clinical studies for COVID-19, but the results were inconclusive [200,209]. Ongoing academic efforts aim to optimise Nsp1 inhibitors with greater potency and specificity.

#### 4.1.6. Challenges and Opportunities

The central challenge is selectively disrupting Nsp1–ribosome binding without perturbing normal translation [210]. Fortunately, the unique C-terminal insertion site offers a specificity window not used by host factors; therefore, identifying specific inhibitors should be achievable [198]. An opportunity lies in the broad-spectrum potential of Nsp1, which is highly conserved among SARS-like coronaviruses belonging to the Sarbecovirus subgenus (which includes SARS-CoV and SARS-CoV-2) [198]. Finally, combining an Nsp1 inhibitor with an antiviral that directly blocks replication could yield additive or synergetic therapeutic benefits [211].

### 4.2. Nsp3 Macrodomain 1 (Mac1)

#### 4.2.1. Biology, Rationale, Assays and Structural Biology

The Mac1 (ADP-ribose-1″-phosphatase) domain of Nsp3, often called a “viral macrodomain”, counteracts host ADP-ribosylation signals and may help the virus evade immune responses involving PARP enzymes [212]. Mac1 is essential for efficient replication and immune evasion, making it a prime target for antivirals [111]. The Mac1 domain’s ADP-ribose binding site has also been determined crystallographically [212,213], and enzymatic assays measuring hydrolysis of ADP-ribose-1″-phosphate allow screening for Mac1 inhibitors [214].

#### 4.2.2. Chemical Matter

**Figure 10:**
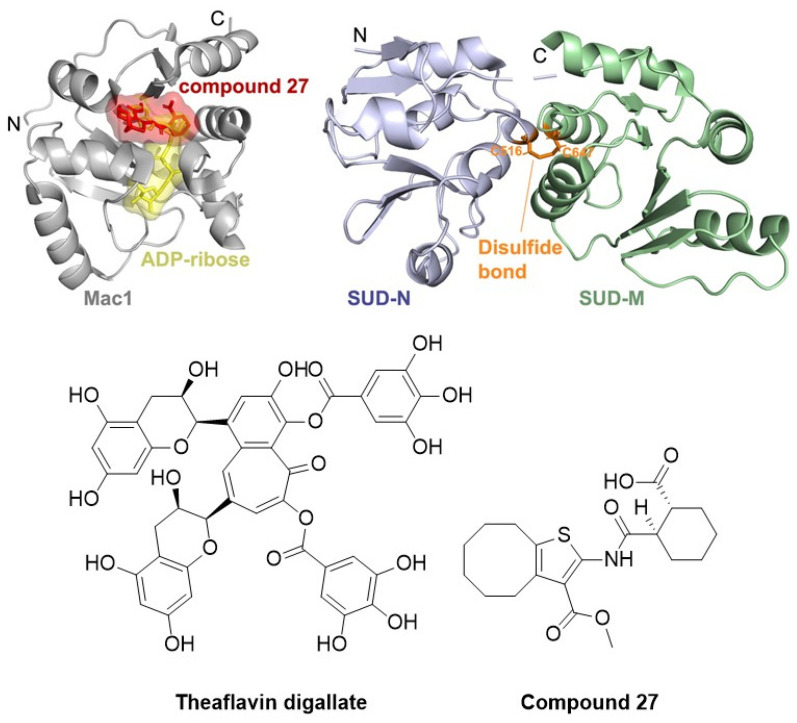
**Top left:** Crystal structure of SARS-CoV-2 Mac1 (grey) in complex with Compound **27** (red; PDB ID 8TV7), with ADP-ribose (yellow; PDB ID 6WOJ) superposed for comparison. **Top right:** Crystal structure of the SARS-CoV-2 SUD containing an engineered disulfide bond (PDB ID 8GQC). The SUD-N domain is shown in purple, the SUD-M domain in green, and the disulfide bond in orange. **Bottom:** Representative chemotypes of SARS-CoV-2 Mac1 and SUD inhibitors, including Compound 27 [216] and theaflavin 3,3′-digallate [109].

Targeting the Mac1 ADP-ribose binding site is challenging, as it is a shallow pocket that binds a small polar substrate [215]. Nonetheless, fragment screening and structure-guided design have identified some hits [215]. A recent study discovered a series of 2-amide-3-methylester thiophene compounds that bind to the Mac1 domain, with one lead compound (Compound **27**, IC_50_ = 2.1 μM) (Figure 10) suppressing coronavirus replication in cells [216]. This provided the first chemical validation of Mac1 as an antiviral target. The Mac1 inhibitor exhibited an on-target mechanism by inhibiting viral RNA replication without significant cytotoxicity, confirming Mac1’s role by showing loss of efficacy against a Mac1-mutant virus [216]. These studies are crucial “hit identification” steps, although Mac1 inhibitors are still in the early phase. Notably, Mac1 is highly conserved in all coronaviruses, including distant ones, so a Mac1 inhibitor could be pan-coronaviral, a strong motivation for further development [217].

#### 4.2.3. Challenges and Opportunities

Mac1 inhibitors would need to avoid human macrodomains, but humans have no close analogue of viral Mac1, lowering the risk of cross-reactivity [218]. Mac1 inhibitor work is more nascent but is attracting interest due to Mac1’s role in suppressing innate immunity and efforts to obtain co-crystal structures of Mac1 with small molecules are ongoing. A current limitation is that inhibitors often have low potency, but fragment-linking approaches or covalent warheads, if a nearby residue allows, may increase affinity. The advantage of this approach is its pan-coronavirus potential. Mac1 is nearly invariant even in distant coronaviruses; therefore, a Mac1 drug could be stockpiled for future coronavirus outbreaks [219].

### 4.3. SARS-Unique Domain (SUD) Within Nsp3

Another interesting and emerging target within Nsp3 is the SARS-unique domain (SUD) [220]. SUD offers an orthogonal mechanism: it binds host/viral G-quadruplex (G4) RNAs and Paip1M, and SARS-CoV-2 SUD is highly flexible, lacking the SARS-CoV interdomain disulfide. Engineering this disulfide back into SARS-CoV-2 was lethal to the virus, underscoring functional constraints alongside conformational plasticity that may complicate design [109]. A biolayer interferometry (BLI)-guided screen identified theaflavin 3,3′-digallate (TF3) (Figure 10) as a direct SUD binder (K_d_ of 2.8 μM) that disrupts SUD–G4 and SUD–Paip1M interactions and shows antiviral activity (Vero E6-TMPRSS2 cells EC_50_ of 5.9 μM and CC_50_ of 98.5 μM; comparable potency in Calu-3 cells), providing proof-of-concept that the SUD–G4 interface harbours exploitable pockets [109]. Together, these data nominate the G4-interface and allosteric patches on SUD as tractable sites, with the caveat that conformational flexibility and the absence of an enzymatic readout demand structure-enabled and biophysics-led campaigns to progress potency and selectivity.

## 5. Cross-Cutting Strategic Lessons for NSP-Directed Antiviral Development

Across the benchmark, near-term, and emerging target tiers discussed above, several strategic lessons have become clear for the continued development of Nsp-directed antivirals. SARS-CoV-2 drug discovery has progressed from an urgent, largely crisis-driven search for deployable single agents to a more mature framework that prioritises mechanistic complementarity, resistance-aware design, and preparedness beyond the current virus. These lessons extend beyond any individual target and instead define the principles that should guide the next generation of antiviral development: how to combine orthogonal mechanisms rationally, how to prioritise conserved viral functions for broader-spectrum utility, and how to integrate structural biology, screening strategies, and pharmaceutical considerations to shorten the path from discovery to clinically useful therapy. In this section, we synthesise these cross-cutting principles to clarify what has been learned from the first wave of SARS-CoV-2 antivirals and which strategic choices are most likely to support durable, variant-resilient, and practically deployable coronavirus treatments in the future.

### 5.1. RTC Coupling as the Mechanistic Basis for Combination Design

**Figure 11:**
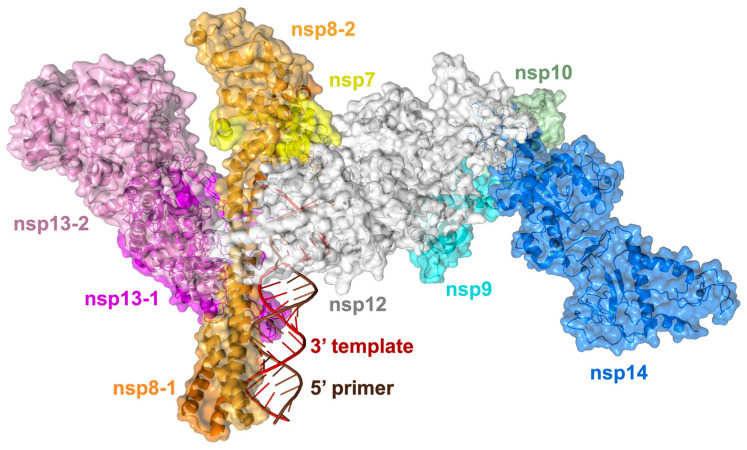
Cryo-EM structure of the SARS-CoV-2 RTC (PDB ID 7EIZ). The template RNA is shown in red, the primer RNA in brown, Nsp7 in yellow, the first copy of Nsp8 in orange, the second copy of Nsp8 in light orange, Nsp9 in cyan, Nsp10 in green, Nsp12 in grey, the first copy of Nsp13 in magenta, the second copy of Nsp13 in pink, and Nsp14 in blue.

SARS-CoV-2 RNA synthesis is executed by the multisubunit RTC (“replisome”) (Figure 11) centred on RdRp (Nsp12) bound to Nsp7 and Nsp8 cofactors. Cryo-EM and crystallography have shown that long helices in Nsp8 form positively charged “sliding poles” that guide nascent RNA and increase processivity. Two helicases (Nsp13) can dock atop the polymerase; their ATP-driven translocation can induce RdRp backtracking, exposing the 3′ end of the nascent strand to the Nsp14 ExoN domain for proofreading and potentially promoting the template-switching events required for subgenomic RNA synthesis. The Nsp10 cofactor is a hub that activates both Nsp14 ExoN and Nsp16 2′-O-MTase, completing mRNA capping along with Nsp14’s N7-MTase activity. Nsp9 contributes to single-stranded RNA binding in the complex. This integrated architecture nicely explains why inhibiting one node can influence others and suggests rational combinations, such as pairing an RdRp inhibitor with agents that target proofreading (ExoN) or capping (MTases), or allosteric disruptors of helicase–polymerase coupling [221].

### 5.2. Combination Strategies and Orthogonal Mechanistic Pairing

Antiviral therapy for coronaviruses is likely to benefit from the same broad principles that transformed HIV and HCV care: targeting independent, essential steps in the viral life cycle and combining agents with orthogonal pathways to improve antiviral effect and prolong durability. For SARS-CoV-2, the strongest current support for this strategy comes from preclinical studies of DAA combinations and from clinical studies pairing antiviral therapy with stage-appropriate immunomodulation.

Preclinically, orthogonal DAA–DAA pairs have shown encouraging results. In vitro studies report consistent synergy between Remdesivir and Nirmatrelvir, and independent work shows that nucleoside analogues can synergise with host-directed entry-pathway inhibitors such as Camostat or Nafamostat [222,223,224]. In addition, combination treatment with Molnupiravir and Nirmatrelvir improved virological and clinical readouts relative to monotherapy in rhesus macaques and improved survival in a lethal mouse model [222,225,226]. Together, these data support the concept that polymerase–protease or virus–host combinations can enhance antiviral efficacy through mechanistic complementarity.

Clinically, the clearest evidence for orthogonal pairing comes from stage-appropriate addition of immunomodulators to antiviral or standard-of-care backbones in hospitalized disease. Baricitinib plus Remdesivir shortened recovery time compared with Remdesivir alone in ACTT-2 [227]. Dexamethasone reduced 28-day mortality in hospitalized patients requiring oxygen or ventilatory support [228]. In critically ill patients, IL-6 receptor blockade with Tocilizumab or Sarilumab improved organ-support-free days and survival, and Baricitinib also reduced mortality in COV-BARRIER [229,230,231]. These data support the broader principle that once disease biology shifts from predominantly viral replication to host inflammatory injury, combining antiviral pressure with calibrated immunomodulation can improve outcomes.

### 5.3. Resistance, Conservation, and Barrier-to-Escape Design

Functional mapping and rapid in vitro selection have delineated multiple resistance pathways for SARS-CoV-2 M^pro^. Deep mutational scanning and serial passage under Nirmatrelvir pressure identified substitutions such as A173V that reduce drug susceptibility while variably impacting fitness, providing a mechanistic atlas of tolerated changes near the S1/S2 subsites [58]. For Ensitrelvir, repeated passage selects M49L, a residue shaping the S2 pocket, frequently in combination with E166A, a key S1 wall contact, with the double mutant displaying marked resistance and a demonstrable fitness cost in drug-free conditions [60]. Orthogonal profiling across protease chemotypes further shows that S144A and L167F confer the greatest resistance to Ensitrelvir, intermediate effects on Nirmatrelvir, and little effect on some next-generation 3CL^pro^ inhibitors, underscoring the value of cross-series benchmarking [232]. Early clinical surveillance is beginning to detect M49L and related changes in treated populations, although at low prevalence to date [233,234].

Resistance to polymerases remains relatively rare in the clinic but is tractable in the laboratory. The Remdesivir-associated E802D substitution in RdRp has emerged in prolonged infections under therapy and carries measurable fitness costs, consistent with the conserved nature of the RdRp active site [96]. Separately, population-scale phylogenomics have linked Molnupiravir use to a distinctive, transition-rich mutational signature (notably G→A changes) in circulating lineages, with occasional onward transmission. This is an expected pharmacodynamic consequence of an error-prone nucleoside, but one that reinforces antiviral stewardship and combination logics [97].

Two principles emerge from this data. First, combination therapy with mechanistically orthogonal agents (e.g., M^pro^ + RdRp) is favoured, as it narrows escape routes and raises the genetic barrier, especially in immunocompromised hosts where viral replication persists. Second, prioritise targets in which resistance carries inherent fitness penalties, such as proofreading and capping machinery, so that any escape demands a virological cost [234].

### 5.4. Discovery-Enabling Technologies: Structural Biology, RTC-Aware Assays, and AI-Assisted Prioritisation

Community-scale, structure-guided discovery during the COVID-19 pandemic showed how rapidly high-quality leads can emerge when crystallography, computation, medicinal chemistry, and open data are tightly integrated. The open-science COVID Moonshot is a prominent example, combining fragment maps, extensive structural data, and iterative design cycles to generate potent non-covalent M^pro^ leads on a rapid timescale [235,236]. Importantly, this effort was distinct from Pfizer’s internal programme that produced Nirmatrelvir, but it nevertheless illustrated a scalable model for accelerated antiviral lead discovery. More recently, ISM3312 has provided a useful example of how generative design, virtual screening, and structural optimisation can be combined to produce a non-peptidic M^pro^ inhibitor with activity against multiple coronaviruses and nirmatrelvir-resistant mutants [48]. Together, these efforts illustrate how rapidly integrated structural, computational, and medicinal-chemistry workflows can accelerate antiviral lead discovery.

In parallel, cryo-EM has illuminated the dynamic states of the RTC. Studies have resolved Nsp13-driven backtracking of RdRp and captured multiple conformational states of the RTC assembly, revealing transient allosteric opportunities that are invisible in static models [237]. Methodological advances, such as time-resolved cryo-EM, are now routinely capturing short-lived intermediates relevant to binding and resistance, tightening the loop between the hypothesis, structure, and design.

### 5.5. Strategic Roadmap for Next-Generation Nsp Antivirals

Despite major therapeutic advances, important scientific and translational gaps still constrain the durability, breadth, and real-world applicability of current antiviral strategies. The next phase of SARS-CoV-2 drug discovery will therefore depend not only on identifying additional active molecules, but on embedding those molecules within a more disciplined development framework that links target choice, treatment timing, resistance management, combination design, delivery, and preparedness. In practical terms, this means prioritising conserved Nsp functions that can support multiple orally deployable antiviral backbones beyond the current M^pro^- and RdRp-centred paradigm, while also recognising that the clinical utility of DAAs is strongly shaped by when and where adequate drug exposure is achieved. Earlier intervention, particularly in high-risk and immunocompromised populations, remains a central objective, and future development programmes should increasingly evaluate not only short-term virologic benefit but also the potential to reduce persistent infection, within-host evolution, and longer-term post-acute sequelae [30,75,238,239].

At the same time, resistance management should become a built-in feature of antiviral development rather than a downstream consideration. This argues for combination-first strategies, prospective genomic surveillance of treatment failure, and prioritisation of targets for which escape is likely to carry meaningful fitness costs. Continued progress will also depend on stronger validation of underexplored Nsp biology, especially where mechanistic promise has not yet been matched by robust structural, cellular, and chemical evidence. Finally, preparedness will require more than target discovery alone: site-of-action optimisation, locally effective delivery where appropriate, stockpile-ready development, equitable access, and sustained inter-outbreak investment in structural biology, screening infrastructure, and rapid design platforms should all be treated as integral parts of next-generation antiviral strategy. Under this framework, the goal is not simply to expand the target list, but to build a durable and translationally credible pipeline of coronavirus therapeutics that remains useful across future outbreaks [58,96,97,240,241].

## 6. Conclusions and Future Directions

In conclusion, coronavirus antiviral discovery has matured over more than two decades of work, built on foundational SARS and MERS research and accelerated by the intensive global effort mobilised during the COVID-19 pandemic. The first generation of DAAs established essential proof of principle: small-molecule inhibition of the SARS-CoV-2 non-structural proteome can rapidly translate from target selection to clinically meaningful therapy. In particular, M^pro^ and RdRp are the benchmark antiviral nodes against which all other SARS-CoV-2 Nsp targets will be judged. Their success validated not only the tractability of coronavirus enzymology but also the broader translational framework required for effective antiviral development, including robust structural biology, fit-for-purpose assay cascades, early pharmacokinetic optimisation, and clinically relevant deployment in outpatient settings.

Simultaneously, the field has moved well beyond the initial protease- and polymerase-centred paradigm. A credible second wave of Nsp targets has now emerged, most notably PL^pro^, Nsp14, Nsp16, Nsp13, and Nsp15, each offering mechanistically distinct opportunities to expand antiviral breadth, strengthen resistance barriers, and diversify future therapeutic strategies. However, the central lesson of the current landscape is that not all tractable targets are equally actionable. From a preparedness perspective, the most promising next-generation programmes should prioritise conserved and assayable non-structural functions supported by strong mechanistic rationale, structural tractability, chemically validated inhibition, reproducible cellular or in vivo antiviral evidence, and realistic prospects for oral, short-course deployment. In that sense, the goal is no longer target expansion for its own sake, but the disciplined progression of the right targets into rational, orthogonal regimens that can improve durability and breadth beyond first-generation single-target approaches.

Looking forward, the future of coronavirus antiviral development will depend on sustained integration rather than isolated research advances. Combination-first thinking, prospective resistance surveillance, RTC-aware assay design, improved delivery and exposure at the relevant site of antiviral action, and continuous investment in structure-guided and computationally enabled discovery should be treated as core components of the antiviral pipeline. If this momentum is maintained, the most enduring legacy of the COVID-19 era will not be a single drug or target class but a durable and reusable development playbook, capable of delivering broadly useful and practically deployable therapeutics against both current SARS-CoV-2 disease and, hopefully, future coronavirus spillover threats. The non-structural proteome remains the strongest foundation for this effort, and its continued prioritisation offers the clearest path toward a truly prepared antiviral landscape.

## Figures and Tables

**Table 1:** Clinically advanced and approved direct-acting antivirals for SARS-CoV-2 targeting M^pro^ or RdRp, with mechanism, route, and regulatory status.

Drug (or Candidate) and Class	Target and Use (Route, Typical Dosing, Start Window)	Key DDIs/Clinical Caveats	Current Status (Region, Date) and Program Notes
**Nirmatrelvir/Ritonavir (Paxlovid)** M^pro^ (3CL^pro^) inhibitor + PK booster	Inhibits SARS-CoV-2 main protease (M^pro^/3CL); oral; 300/100 mg q12h × 5 days; start ≤5 days from symptom onset	Many DDIs via strong CYP3A inhibition (Ritonavir); avoid strong CYP3A inducers; check all meds; renal dose adjustment (eGFR 30–59)	Approved US (May 2023); Full MA EU (February 2023); special emergency approval JP (2022); conditional approval CN (2022)
**Remdesivir (Veklury)** RdRp nucleoside analogue (IV)	Inhibits viral RdRp; IV outpatient: 200 mg day 1 to 100 mg days 2–3; start ≤7 days	No major CYP DDIs; do not co-administer Chloroquine/Hydroxychloroquine (antagonism)	Approved US (October 2020); full MA EU (August 2022); approved JP (May 2020)
**Molnupiravir (Lagevrio)** RdRp mutagenesis (oral)	Error catastrophe in viral RNA; oral 800 mg q12h × 5 days; start ≤5 days	Minimal identified CYP DDIs; avoid in pregnancy	EUA US (not full approval); application withdrawn EU after negative CHMP (2023); authorised by MHRA (GB)
**Ensitrelvir (Xocova)** M^pro^ (3CL^pro^) inhibitor (oral)	Inhibits M^pro^; oral Day 1: 375 mg; Days 2–5: 125 mg QD; start preferably ≤72 h (trials ≤3–5 d)	CYP3A inhibitor; may increase exposure to CYP3A substrates such as Midazolam and Tacrolimus.	Full approval Japan (March 2024); global development ongoing (e.g., PEP)
**Simnotrelvir/Ritonavir (Xiannuoxin)** M^pro^ (3CL^pro^) inhibitor + PK booster	Inhibits M^pro^; oral 750 mg Simnotrelvir + 100 mg ritonavir q12h × 5 days; start ≤3 days (pivotal)	Ritonavir-mediated CYP3A DDIs (similar to Paxlovid)	Regular approval China (July 2024)
**Leritrelvir (RAY1216)** M^pro^ (3CL^pro^) inhibitor (oral)	Inhibits M^pro^; oral 400 mg TID × 5 days (local label); early treatment (≤~5 d in trials)	Fewer PK DDIs vs. Ritonavir-boosted regimens; verify local label	Conditional approval China (March 2023)
**Deuremidevir HBr (VV116)** RdRp nucleoside prodrug (oral)	GS-441524-like analogue (RdRp); oral Day 1: 600 mg q12h; Days 2–5: 300 mg q12h; start ≤5 days	Low CYP DDI potential expected	Conditional approval China (January 2023)
**Azvudine (FNC)** Nucleoside analogue (oral)	RdRp inhibition (± immunomodulation); oral 5 mg QD up to ≤14 days; early mild–moderate disease	Minimal CYP involvement; monitor with certain concomitants (e.g., anticoagulation)	Conditional approval China (July 2022)
**Obeldesivir (GS-5245)** RdRp nucleoside prodrug (oral)	Oral prodrug of GS-441524 (RdRp); ≈350 mg q12h × 5 days; start ≤5–7 days	Low DDI potential (not a strong CYP/P-gp modulator in early data)	Phase 3 programs with antiviral effect but mixed clinical outcomes; not approved
**Bemnifosbuvir (AT-527)** RdRp nucleoside (oral)	Protocol-dependent dosing; start typically ≤5 days	Low expected DDI burden	SUNRISE-3 Phase 3 showed limited benefit; program deprioritized; not approved
**Pomotrelvir (PBI-0451)** M^pro^ (3CL^pro^) inhibitor (oral)	Ritonavir-free design; dosing not advanced to label-like regimen	—	Phase 2 topline (April 2023) program suspended; not approved
**EDP-235** M^pro^ (3CL^pro^) inhibitor (oral)	Dosing not advanced to label-like regimen	—	Phase 2 mixed results; program effectively paused; not approved

(a) Scope and caveats. Adult with mild-to-moderate COVID-19 unless otherwise stated. “Start window” = time from the symptom onset. Dosing may require renal/hepatic adjustment; confirm pregnancy/paediatric use and contraindications with the local SmPC/PI. DDIs listed are non-exhaustive. Ritonavir is a strong CYP3A inhibitor (watch narrow therapeutic index substrates/inducers). (b) Regulatory legend. Status current as of March 2026. US = FDA; EU = EMA (MA = Marketing Authorisation); JP = PMDA (including special emergency approvals); CN = NMPA; EUA = Emergency Use Authorization; PEP = post-exposure prophylaxis; MHRA = Medicines and Healthcare products Regulatory Agency. (c) Abbreviations. 3CL^pro^/M^pro^ = main protease; RdRp = RNA-dependent RNA polymerase; QD = once daily; BID = twice daily; TID = three times daily; q12h = every 12 h; IV = intravenous; eGFR = estimated glomerular filtration rate. (d) Inclusions. Small-molecule antivirals only (<1500 Da); biologics (e.g., mAbs), immunomodulators, and non-antiviral therapies were out of scope.

## Data Availability

No new data were created or analyzed in this study.

## Abbreviations

The following abbreviations are used in this manuscript:

2′-O-MTase	2′-O-methyltransferase
3CL^pro^	3C-like protease (Nsp5)
ACTT	Adaptive COVID-19 Treatment Trial
ADME	Absorption, distribution, metabolism, and excretion
AI	Artificial intelligence
ATA	Aurintricarboxylic acid
ATP	Adenosine triphosphate
BID	Twice daily
BLI	Biolayer interferometry
CC_50_	50% cytotoxic concentration
CFA	Chlorofluoroacetamides
CHMP	Committee for Medicinal Products for Human Use
CN	National Medical Products Administration of China (NMPA)
CoV	Coronavirus
COVID-19	Coronavirus Disease 2019
Cryo-EM	Cryo-electron microscopy
CYP	Cytochrome P450
CYP3A	Cytochrome P450 3A
DAA(s)	Direct-acting antiviral(s)
DDI	Drug–drug interaction
DMPK	Drug metabolism and pharmacokinetics
DNA	Deoxyribonucleic acid
DUB	Deubiquitinase
EC_50_	Half-maximal effective concentration
eGFR	Estimated glomerular filtration rate
EMA	European Medicines Agency
EPIC-HR	Evaluation of Protease Inhibition for COVID-19 in High-Risk Patients
EU	European Union/European Medicines Agency (EMA)
EUA	Emergency Use Authorization
ExoN	Exoribonuclease (Nsp14)
FBDD	Fragment-based drug design
FP	Fluorescence polarization
FRET	Fluorescence resonance energy transfer
G4	G-quadruplex
GI	Gastrointestinal
HCV	Hepatitis C virus
HDX-MS	Hydrogen–deuterium exchange mass spectrometry
hERG	Human ether-à-go-go-related gene potassium channel
HIV	Human immunodeficiency virus
HTS	High-throughput screening
IC_50_	Half-maximal inhibitory concentration
IFIT	Interferon-induced protein with tetratricopeptide repeats
IFN	Interferon
IL	Interleukin
ILR	Interleukin receptor
IRF3	Interferon regulatory factor 3
ISG	Interferon-stimulated gene
ISG15	Interferon-stimulated gene 15
IV	Intravenous
JAK	Janus kinase
JP	Pharmaceuticals and Medical Devices Agency of Japan (PMDA)
K_d_	Equilibrium dissociation constant
MA	Marketing Authorisation
mAbs	Monoclonal antibodies
Mac1	Macrodomain (of Nsp3)
MDA5	Melanoma differentiation-associated protein 5
MERS-CoV	Middle East Respiratory Syndrome Coronavirus
MHRA	Medicines and Healthcare products Regulatory Agency
MHV	Mouse hepatitis virus
M^pro^	Main protease (Nsp5)
mRNA	Messenger RNA
MST	Microscale thermophoresis
MTase	Methyltransferase
N7-MTase	N7-methyltransferase (Nsp14)
NendoU	Endoribonuclease (Nsp15)
NHC-TP	N4-hydroxycytidine triphosphate
NiRAN	Nidovirus RdRp-associated nucleotidyltransferase (N-terminal domain of Nsp12)
NMPA	National Medical Products Administration (China)
NMR	Nuclear magnetic resonance
Nsp(s)	Non-Structural Protein(s)
NTP	Nucleoside triphosphate
ORF	Open reading frame
PARP	Poly(ADP-ribose) polymerase
PDB	Protein Data Bank
PEP	Post-exposure prophylaxis
P-gp	P-glycoprotein
PK/PD	Pharmacokinetics/pharmacodynamics
PL^pro^	Papain-like protease (Nsp3)
PMDA	Pharmaceuticals and Medical Devices Agency (Japan)
pp1a	Polyprotein 1a
pp1ab	Polyprotein 1ab
PPI	Protein–protein interaction
q12h	Every 12 h
QD	Once daily
qPCR	Quantitative polymerase chain reaction
RdRp	RNA-dependent RNA polymerase (Nsp12)
RECOVERY	Randomised Evaluation of COVID-19 Therapy
REMAP-CAP	Randomised, Embedded, Multifactorial Adaptive Platform Trial for Community-Acquired Pneumonia
RNA	Ribonucleic acid
RTC	Replication-transcription complex
SAH	S-adenosyl-L-homocysteine
SAM	S-adenosylmethionine
SAR	Structure–activity relationship
SARS-CoV	Severe Acute Respiratory Syndrome Coronavirus (2002–2003)
SARS-CoV-2	Severe Acute Respiratory Syndrome Coronavirus 2
SBDD	Structure-based drug design
SF	Superfamily
SPA	Scintillation proximity assay
ssRNA	Single-stranded RNA
SUD	SARS-unique domain (of Nsp3)
TBK1	TANK-binding kinase 1
TID	Three times daily
TMPRSS2	Transmembrane protease, serine 2
TWNK	Twinkle helicase
US	United States Food and Drug Administration (FDA)
USP13	Ubiquitin-specific peptidase 13
